# Highly Branched Tannin-Tris(2-aminoethyl)amine-Urea Wood Adhesives

**DOI:** 10.3390/polym15040890

**Published:** 2023-02-10

**Authors:** Bengang Zhang, Xinyi Chen, Antonio Pizzi, Mathieu Petrissans, Stephane Dumarcay, Anelie Petrissans, Xiaojian Zhou, Guanben Du, Baptiste Colin, Xuedong Xi

**Affiliations:** 1 LERMAB, IUT Hubert Curien, University of Lorraine, 7 rue Fusillés Résistance, 88000 Epinal, France; 2 LERMAB-ENSTIB, University of Lorraine, 27 rue Philippe Seguin, 88000 Epinal, France; 3 Yunnan Key Laboratory of Wood Adhesives and Glue Products, College of Material Science and Engineering, Southwest Forestry University, Kunming 650224, China; 4 LERMAB, Faculté des Sciences, University of Lorraine, Blvd. des Aiguillettes, 54000 Nancy, France

**Keywords:** tannin, tris(2aminoethyl)amine, urea, copolymerized networks, wood adhesives, thermoset resins, wood panels, MALDI ToF, ^13^C NMR

## Abstract

Condensed tannin copolymerized with hyperbranched tris(2-aminoethyl)amine-urea formed by amine-amido deamination yields a particleboard thermosetting adhesive without any aldehydes satisfying the requirements of relevant standards for the particleboard internal bond strength. The tannin–triamine–urea cures well at 180 °C, a relatively low temperature for today’s particleboard hot pressing. As aldehydes were not used, the formaldehyde emission was found to be zero, not even in traces due to the heating of wood. The effect is ascribed to the presence of many reactive sites, such as amide, amino, and phenolic groups belonging to the three reagents used. The tannin appears to function as an additional cross-linking agent, almost a nucleating agent, for the triamine–urea hyperbranched oligomers. Chemical analysis by MALDI ToF and ^13^C NMR has shown that the predominant cross-linking reaction is that of the substitution of the tannin phenolic hydroxyls by the amino groups of the triamine. The reaction of tannin with the still-free amide groups of urea is rather rare, but it may occur with the rarer tannin flavonoid units in which the heterocyclic ring is opened. Due to the temperature gradient between the surfaces and the board core in the particleboard during hot pressing, the type and the relative balance of covalent and ionic bonds in the resin structure may differ in the surfaces and the board core.

## 1. Introduction

The wood composites industry is mainstream in the world’s wood processing industry. In recent years, people’s awareness of green environmental protection has been heightened day by day. This, coupled with the increasing scarcity of fossil resources such as oil and natural gas, has prompted researchers to focus, among others, on developing biosourced resins for wood adhesives without formaldehyde. An example is protein-based sea mussel adhesives. These proteins, which are rich in catechol groups, yield a formaldehyde-free adhesive derived from renewable resources. As sea proteins for such an adhesive protein are not easily available, other catechol-rich biomaterials such as flavonoid tannins have been used for an adhesive system. Thus, mixtures of proanthocyanidin-type condensed tannins and polyimine (PEl) were found to be an excellent substitute for marine adhesion proteins [1] as wood adhesives without any formaldehyde. The bonding of wood composites with this adhesive presented good shear strength and water resistance.

A new concept has more recently been proposed to synthesize urea-based hyperbranched polymers (HBPs) using polyamines and urea, and the strategy used is to directly use the synthesized HBPs to bond plywood [2]. The reaction of amines and polyamines with urea is well known and well codified [3,4,5,6,7]. For the adhesive in reference [2], during hot pressing, the HBPs further condense and solidify, resulting in a formaldehyde-free wood adhesive with good bonding properties [2]. Urea is used for this synthesis because it is very inexpensive. Structural characterization indicated that the cured structure of the polymer proposed as a binder presented amine and urea terminal groups with their respective proportions, depending on the ratio of monomers used. This resin also exhibited excellent water resistance and bond strength. However, this approach was used for bonding plywood by using far too high of a curing temperature (189–220 °C) for such a type of panel; the temperature should be around 120 °C. This seems to indicate that such a resin curing rate may be highly temperature sensitive and thus less suitable for adhesives for plywood applications, but it may be more useful for a different type of assembly, such as an adhesive for wood particleboard for which the press temperature used industrially is much higher, perhaps even without the addition of a curing accelerator.

However, due to the high cost of directly using synthetic HBPs as an adhesive for wood-based panels, it would be of interest to rather consider it to be just used as a modifier for wood particleboard adhesives. The coupling of different types of hyperbranched polymer networks to wood adhesives to improve their performance has been a recent line of investigation for quite some time. Thus, the addition of hyperbranched polymers to urea–formaldehyde resins [8,9,10], to melamine and melamine urea–formaldehyde adhesives [11,12,13], and even to tannin resins [14] are a known as a functional approach to upgrade these resins’ performances.

**Figure 1:**
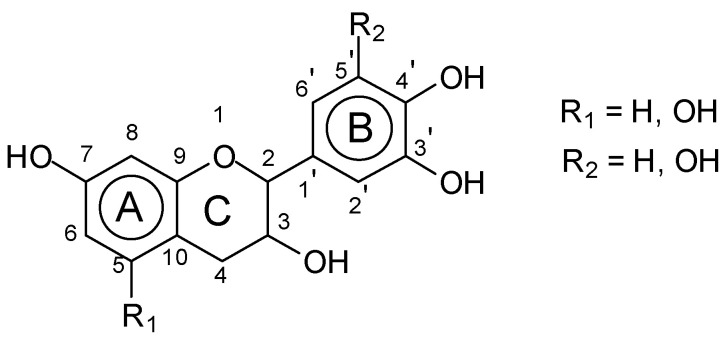
Basic structure of a condensed tannin flavonoid unit.

Condensed tannins are highly reactive chemical species [15,16] that have already been commercialized for wood panel adhesives, although by using formaldehyde and other aldehydes as hardeners [17,18,19]. They are constituted of flavonoid units of four types (Figure 1) 

This means that condensed tannins could well function as strong accelerators of triamine–urea hyperbranched networks. By modifying with tannins, a relatively inexpensive triamine–urea hyperbranched resin, the bond strength and water resistance of non-aldehyde tannin-based adhesives should be markedly improved. The substitution reactions of the phenolic -OH groups of tannin by ammonia [20] and of tannins and lignin by amine -NH_2_ groups are well known and studied in depth [21,22]. The present work thus presents such an approach for tannin adhesives. This work thus reports a copolymerized tannin–triamine–urea adhesive for particleboard. The novelties of this approach for hyperbranched adhesives are multiple: (i) the increase in the percentage of biomaterials of up to and more than 90%, taking into account both the urea and the tannin intended for this use in hyperbranched-based wood adhesives for particleboard, (ii) the elimination of formaldehyde or any other aldehyde from the adhesive, (iii) the elimination of traces of formaldehyde generated by the heating of wood [23] and by reaction with all the residual amide, amine, and phenolic sites, and (iv) the preparation of an adhesive presenting good bonding results. In this last context, it is necessary to point out that the high reactivity of tannins functions as an accelerator of curing of the whole adhesive. In particleboards, while the surfaces are at the temperature of the hot press plates, between 180 °C for older factories and 220 °C for more modern ones, the core part of the panel never reaches temperatures higher than 115–120 °C at the industrial press times used [24,25]. Thus, the addition of the tannin would allow for the use of hyperbranched wood adhesives at a much lower temperature than the one reported for plywood from previous work [2].

## 2. Materials and Methods

### 2.1. Materials

Commercial mimosa tannin extract (Acacia mearnsii, De Wild) was supplied by Silva Chimica (St. Michele Mondovi, Italy). Urea (99.5%, ACS reagent, Sigma-Aldrich, St. Louis, MO, USA). Distilled water (lab-made). Tris(2-aminoethyl)amine (96%, Sigma-Aldrich). Catechin (Sigma-Aldrich). Sodium hydroxide and hydrochloric acid were also used. All chemical reagents did not need purification before use. 

### 2.2. Preparation of 50% Solution of Highly Branched Polymer (HBP)

Tris(2-aminoethyl)amine:Urea (U) = 1:1.5 (molar ratio) preparation: 14.6 g of tris(2-aminoethyl) amine and 9 g of U [2] were reacted in an oil bath at 115 °C for 8 h. Distilled water was then added after the reaction to obtain a 50% HBP solution after complete dissolution. This preparation was repeated several times to prepare a sufficient amount of HBP solution for the experiments that followed. 

### 2.3. HBP-Modified Tannin Resin

The tannin extract was dissolved in water to form a smooth 50% solution, which is recorded as T0. The HBP was then added in the proportions indicated in Table 1.

### 2.4. Viscosity and Solid Content

Viscosities were measured with a rotary Brookfield RV viscometer (AMETEK Brookfield, Middleboro, MA, USA) using different spindles and different rotation rates. The resins’ solid contents were tested by placing about 1 g of resin in a small Petri dish and drying it in an oven at 103 °C overnight. The samples were weighed before and after drying. The test was repeated three times, and the results were averaged.

### 2.5. Particleboard Preparation and Testing

An appropriate amount of 50% tannin solution and highly branched polymer solution were blended uniformly at room temperature, and the pH was adjusted to 10 to test the viscosity and solid content, and then, three panels of one-layer laboratory particleboard of 400 × 400 × 14 mm for each resin tested were prepared. The moisture content of the resinated wood chips was 9.7%, the resin solid load applied to the wood chips was of 10% on dry wood, and the aimed target density of the particleboard was 0.700 g/cm^3^. The boards were pressed to gauges at a temperature of 180 °C at a maximum pressure of 28 kg/cm^2^ for a pressing cycle of 7 min. After cooling, the particleboard panels were then tested for bending strength, internal bond (IB) strength, overall density according to the European norm EN312-2010 [26], and thickness swelling after 2 h and 24 h in cold water; the latter two were just for a comparative test. It must be pointed out that no wax emulsion to improve water repellence was added to the panels, and the panels were composed of one single layer of industrial core wood chips that would give higher swelling to the panel in water. The particleboards prepared were tested also for formaldehyde emissions. All the cases did not yield any formaldehyde emissions, not even traces that could have been reasonable to expect from the heating of the wood, as even these traces would be absorbed and neutralized by the mass of free reactive sites, both the amino and amide groups, and the tannin phenolic sites.

### 2.6. Thermogravimetric Analysis (TGA) 

The samples were tested for their temperature stability characteristics by a thermogravimetric analyzer (NETZSCH, STA 449 F3 Instruments, Weimar, Germany). In each run, around 5–10 mg of sample was loaded into an Al_2_O_3_ crucible, and then, the crucible was placed into the TGA furnace. N_2_ at a flow rate of 100 mL/min was used as the carrier gas. In the TGA, the raw or biochar samples were heated from 25 °C at a heating rate of 20 °C/min to 800 °C.

### 2.7. Thermomechanical Analysis (TMA)

A total of 30 mg of the prepared adhesive was applied to two beech wood chips, with dimensions of 17 × 5 × 1.1 mm^3^, stacked together into two-ply samples. The relationship of the modulus of elasticity (MOE) against temperature was recorded by TMA (Mettler Toledo 40, Zurich, Switzerland) following the method of three-point bending at a heating rate of 10 °C/min in the range 30–250 °C, and the relevant curves of the modulus of elasticity (MOE) against temperature were registered.

### 2.8. Fourier Transform Infrared (FT-IR) Spectroscopy

A FTIR/NIR spectrometer with attenuated total reflection (ATR) (ATR cell on a Perkin Elmer Spectrum 2000, Perkin Elmer France, Villebon-sur-Yvette, France) was used to analyze the chemical structure of the melamine–dialdehyde starch resin. The FTIR spectra consisted of 32 scans collected per run with a spectral resolution of 4 cm^−1^ at room temperature in the 4000–650 cm^−1^ range; each sample was tested 10 times.

### 2.9. Matrix-Assisted Laser Desorption Ionisation Time of Flight Mass Spectrometry (MALDI ToF)

The samples (4 mg/mL) were dissolved in water/acetone (50/50 by volume), and a 10 mg/mL matrix solution in acetone was added. To facilitate placing the samples on the sample holding plate, the matrix used was 2,5-dihydroxy benzoic acid. The spectrometer was calibrated by using red phosphorus (LaserBio Labs, Valbonne, France). Sodium chloride (NaCl) was added to the matrix as a distilled water 10 mg/mL solution to maximize ion formation. This was conducted as NaCl added to the matrix facilitates the flight of heavier oligomers in the spectrometer. Once the sample was added to the matrix solution, the mixture was divided into three separate portions of the solution. These three portions of the sample/matrix solution plus 0.5 to 1 µL of NaCl solution were placed on the MALDI on three separate targets of the holding plate. The solvent was then evaporated, and then, the sample target holder was put in the spectrometer. The spectrum peaks can either represent the actual molecular weight of the molecular species detected or represent the molecular weight plus the Na+ molecular weight (23 Da) linked to the molecule, derived from the NaCl added to the matrix. Occasionally, the same chemical species can yield peaks with and without Na+ in the same spectrum. An AXIMA Performance MALDI ToF (Shimadzu Scientific Instruments; Manchester, UK) spectrometer was used. The irradiation source was a pulsed nitrogen laser with 3-ns intervals at a wavelength of 337 nm. The conditions used were positive polarity, linear flight path, high mass (20-kV accelerating voltage), and 100 to 150 pulses per spectrum. Delay times of 200 to 800 ns were used by the application of the delayed technique. The ion gate was set at 0 Da, 500 Da, 1000 Da, and 1500 Da, respectively. The spectra are exact at ±1 Da.

### 2.10. CP MAS ^13^C NMR

First, catechin (0.5 g) was used as the model compound of a flavonoid tannin. A total of 0.5 g of hexamethylene diamine (HMDA) was then mixed with it as a 70% solution in water followed by 0.15 g of NaOH 33 wt % water solution. The mixture was then reacted at 100 °C in an oven. Solid-state CP-MAS (cross-polarisation/magic-angle spinning) ^13^C NMR spectra of the aforementioned oven-dried solids were obtained on a Brüker MSL 300 spectrometer (Brüker France, Wissembourg, France) at a frequency of 75.47 MHz. Tetramethyl silane (TMS) was used to calculate the chemical shifts. The conditions used were 4 kHz rotor spinning on a double-bearing 7 mm Bruker probe. Recycle delays were 5 s with a 90° pulse. The spectra were acquired with 5 s recycle delays, a 5 s 90° pulse, and a contact time of 1 ms for 3000 transients. 

Second, mimosa tannin extract was reacted with the tris(2-aminoethyl)amine-urea hyperbranched polymer at 100 °C in an oven, and solid-state CP-MAS (cross-polarisation/magic-angle spinning) ^13^C NMR spectra were obtained on a Brüker MSL 300 spectrometer (Brüker France, Wissembourg, France) using the solids obtained by drying. The frequency used was 75.47 MHz. Tetramethyl silane (TMS) was used as the base for calculating the spectra chemical shifts. The conditions used were 4 kHz rotor spinning on a double-bearing 7 mm Bruker probe, 5 s recycle delays, a 90° pulse of 5 s; and a contact time of 1 ms for 3000 transients.

## 3. Results and Discussion

### 3.1. TMA

**Figure 2:**
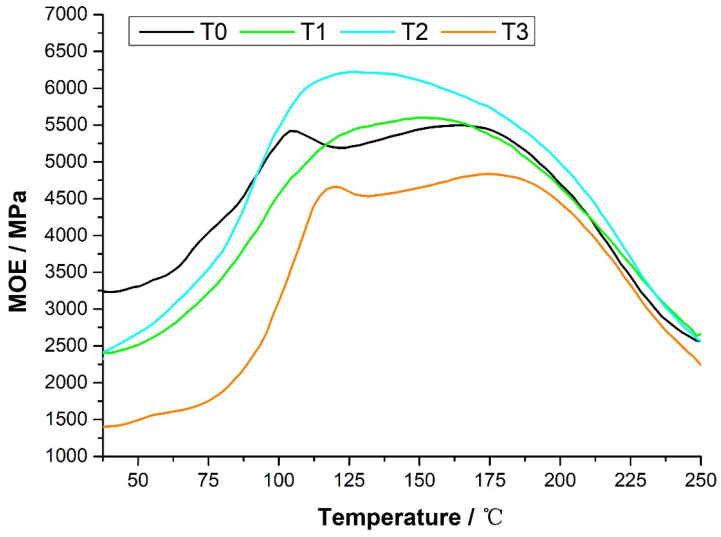
TMA curve of the highly branched polymer-modified tannin resin.

Figure 2 shows the TMA temperature profiles for T0, T1, T2, and T3, showing three important regions related to the MOE change in the adhesive during curing. The first region of T0, T1, T2, and T3 is 35.8–89.9 °C, which is the low MOE stage. At this stage, the adhesives behave as liquids, and they cannot transmit stress between the wood layers. At this stage, the order of the MOE of the adhesive is T0 > T2 > T1 > T3.

The second region is 89.9–116.2 °C, where the MOE increases with increasing temperature, indicating that the adhesive gradually changes from liquid to rubbery and solid. At this stage, the order of the MOE of the adhesive is T2 > T0 > T1 > T3.

The third region is 116.2–181.7 °C, where the MOE of the adhesive increases with temperature to the highest level, and bonds are formed. At this stage, the highest MOE values of adhesives T0, T1, T2, and T3 were 5497.55, 5601.39, 6221.25, and 4834.88 MPa, respectively, the temperatures at which the highest MOE values were reached were 163.6, 149.3, 127.2, and 174.4 °C, and the order of the highest MOE values was T2 > T1 > T0 > T3, indicating that the rate of reaction to complete curing was more rapid for T2 and T1.

After reaching the maximum value, the MOE of the adhesives T0, T1, T2, and T3 all decreased, this being due to the thermal degradation process of the adhesive.

### 3.2. TGA/DTG

**Figure 3:**
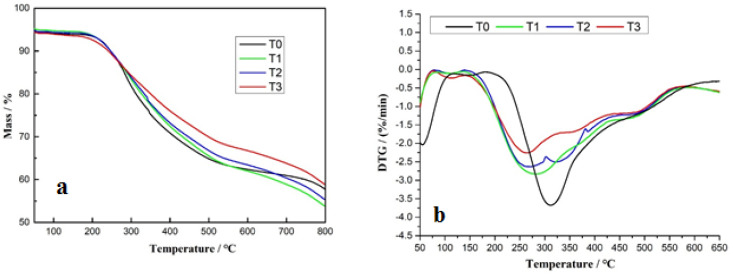
TG curves (**a**) and DTG (**b**) of T0, T1, T2, and T3 resins in the 50–650 °C temperature range.

To investigate the thermal stability of the fully cured series of resins, thermogravimetric analysis (TGA) was carried out. The corresponding resulting TGA curves, and particularly the first derivate (DTG), are shown in Figure 3a,b. From the DTG curve, there appears to be several stages in the thermal degradation process, thus, for T1, T2, and T3 in the ranges of 50–125 °C, 175–300 °C, and three zones between 300–600 °C. For the first in the lower temperature range, the weight loss can be attributed to residual water evaporation and small molecular impurity degradation [27,28]. The second range corresponds to degradation of the urea and its linkages with the triamine. The series of small stages between 350 °C and 600 °C correspond to the progressive degradation of the materials of the network formed, first the triamine and its linkages, and last, the degradation of the tannin molecules; condensed tannins degrade at high temperatures as generally they are used for improving the temperature resistance of resins and adhesives. Some more stable chemical bonds, such as C-C and C-O, are cleaved within this temperature range [27,28,29].

### 3.3. Properties of Particleboard

#### 3.3.1. Viscosity and Solid Content

The materials prepared are all thixotropic, the same behavior being characteristic of the colloidal state of tannin in the solution itself. Since the viscosity value is always changing when testing the viscosity, the range value is taken after the change and once the viscosity tends to stabilize (Table 2).

#### 3.3.2. Mechanical Properties

The average results of the particleboard tests are shown in Table 3. It can be seen from the table that the results of T1, T2, and T3 yield significantly better bending strength and internal bond (IB) strength results than the T0 control. Conversely, the differences between T1, T2, and T3 are not major, indicating that even a relatively low proportion of hyperbranched triamine–urea polymer considerably upgrades the performance of the tannin adhesive, and conversely, the tannin improves the results of the exclusively hyperbranched triamine–urea adhesive described previously [2].

### 3.4. FTIR

**Figure 4:**
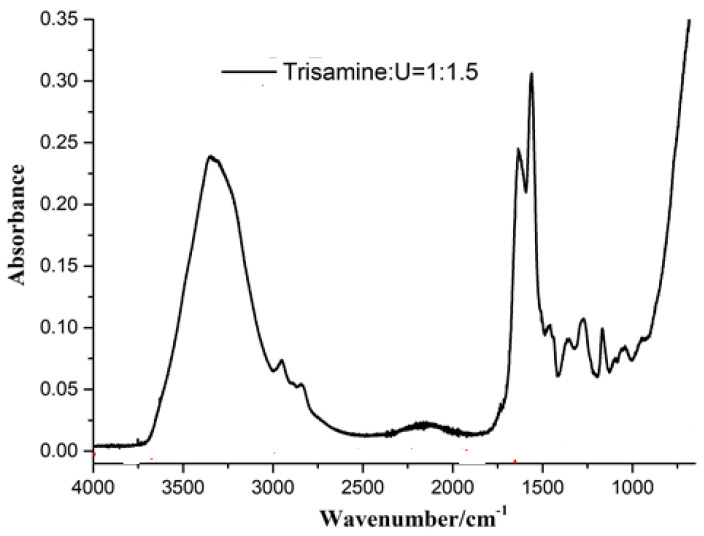
FTIR of the hyperbranched reaction product of tris (2–amino ethyl) amine with urea.

The FTIR spectrum of the hyperbranched tris(2-aminoethyl)amine-urea polymer is shown in Figure 4. The very wide peak centered at 3250 cm^−1^ is representative of the peak of the -CO-NH-C stretch of reacted amide groups and of urea that is covered by the asymmetric stretching of urea (H2N-CO-NH_2_), the three peaks of which are present in the 3500–3100 cm^−1^ range [29,30]. Hidden by this wide peak are also the asymmetric stretching peaks of the primary amide groups (-CO-NH_2_) and the primary amine (-CH-NH_2_) asymmetric stretching that absorbs between 3400 and 3330 cm^−1^ [29,30]. While the small but sharp peak at 1190 cm^−1^ confirms again the presence of reacted amide groups, the very small peak at 1100 cm^−1^ belongs also to the asymmetric stretching of the unreacted amine groups and confirms the presence of still unreacted primary amine groups in the hyperbranched polymer [29,30]. The two small peaks at 2930 and 2840 cm^−1^ are assigned to the alkyl chains (R-CH_2_-R′) of the triamine. The low but wide band between 2000 and 2200 cm^−1^ is assigned to -NH_2_^+^ [29,30]. The sharp doublet of peaks at 1650 cm^−1^ and 1540 cm^−1^ confirms the reaction of urea as it is assigned to the -C=O of the substituted urea and to the stretching of the reacted urea groups (-CO-NH-C-), this being confirmed by the small band at 1412 cm^−1^ [29,30]. The small peak at 1343 cm^−1^ is assigned to the -N= peak of the tertiary amine, and the 1303 cm^−1^ peak corresponds to the protonate -NH_3_^+^ groups stretching [29,30].

**Figure 5:**
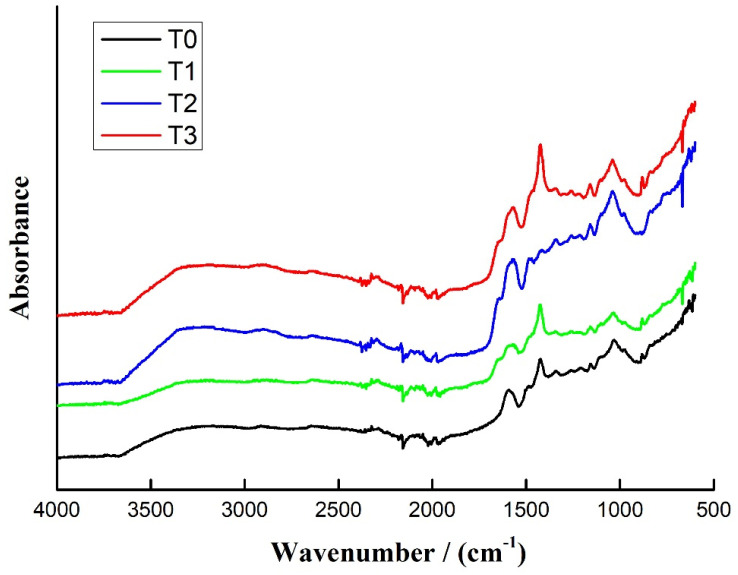
FTIR spectra of the highly branched polymer-modified tannin resins.

The FTIR spectra of T0, T1, T2, and T3 are shown in Figure 5. It can be seen from the figure that these spectra have a broad peak at 3405–3113 cm^−1^, which is the characteristic peak of hydroxyl (-OH) [31], secondary amides, -NH- stretching, etc. The small peak near 2914 cm^−1^ is caused by the aromatic C-H stretching vibration [32]. The absorption peak near 1686 cm^−1^ is assigned to the C=O of amides [33] and the peak at 1604 cm^−1^ to the tertiary amine group -N= of the triamine. The absorption peak at 1352 cm^−1^ belongs to the -NH_2_ of the amine; the absorption peak at 1262 cm^−1^ is the amine C-N elongation [34]. Not much can be gleaned from just the FTIR of the four resins.

### 3.5. MALDI ToF

**Figure 6:**
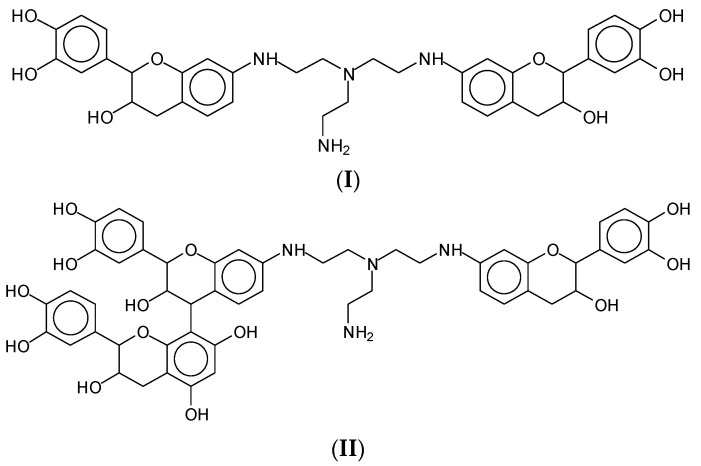

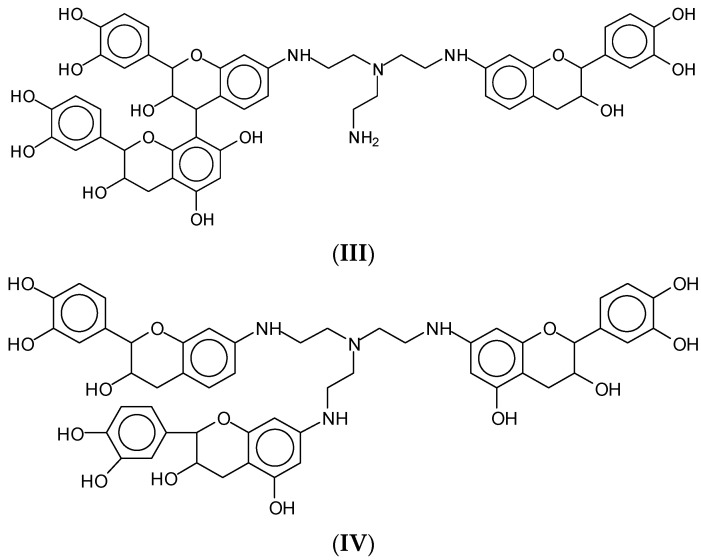
Chemical species (**I**–**IV**) are formed by the reaction of flavonoid units with the free triamine noy yet involved in the hyperbranched network.

The formation of hyperbranched polymers from the reaction of tris(2-aminoethyl)amine deamination with urea is known [2]. However, it is of importance to consider the series of oligomers formed, leading to the progressive formation of hyperbranched polymers of this type (Figure S1, Table S1, Supplementary Material). First of all, it needs to be pointed out that tris(2-aminoethyl)amine can undergo the reaction of substitution of the phenolic hydroxyl groups of tannin by itself, without forming the hyperbranched triamine–urea structure. Thus, species such the ones at 551 Da (**I**), 657 Da (**II**), and 945 Da (**III** and **IV**) (Figure 6) are also formed in the reactions of flavonoid units with the still available triamine species not yet involved in the hyperbranched network (Table S2, Figure S2 Supplementary Materials).

This means that the hyperbranched structure can, in principle, form also by just reacting tris (2-aminoethyl)amine with the tannin oligomers, but the addition of urea preforming, however, is a hyperbranched network that is more rapid to set and harden once the tannin is added.

**Figure 7:**
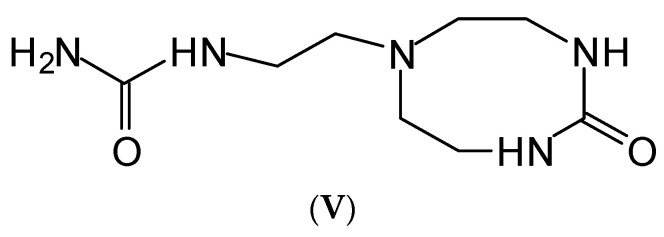
The only cyclic structure detected of the reaction of triamine with urea.

Of the two cyclic reaction products cited by other authors [2] for the reaction of tris(2-aminoethyl)amine-urea, only the one at 216 Da has been observed in minor amounts (Figures S1 and S2, Tables S1 and S2, Supplementary Materials) (Figure 7) (**V**).

**Figure 8:**
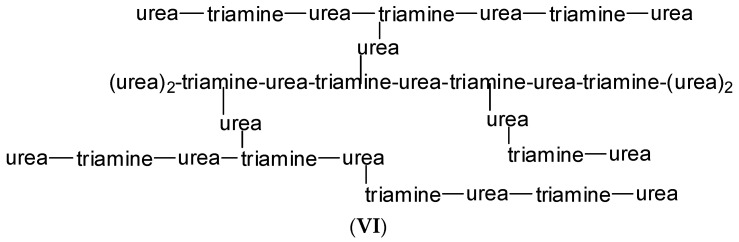
A higher molecular weight structure of the tramine-ureahyperbranched network.

The MALDI ToF investigation of the first part of this reaction has shown the progressive reaction of urea and tris (2-aminoethyl) amine proceeding from small oligomer species to more complex structures. The MALDI ToF spectra and the list of the species identified are shown in Figure S1 and Table S1 in the Supplementary Materials. Here, it is sufficient to indicate the complex structures up to a molecular weight of 2456 Da; these have been identified as structure (**VI**) (Figure 8).

Even higher molecular weight hyperbranched structures are formed by the continued addition of tris(2-aminoethyl)amine-urea units to the already sizeable tridimensional oligomers. 

**Figure 9:**
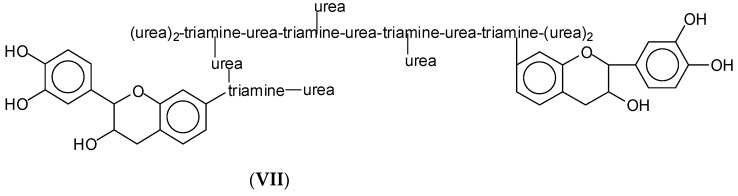
An example of a detected structure with two flavonoid units linked to a triamine-urea hyperbrached network part.

Of interest here is instead the reaction of a condensed flavonoid tannin with the oligomers of the hyperbranched tris(2-aminoethyl)amine-urea to form a workable wood panel adhesive without the use of any aldehyde. Several interesting species are formed (Figure S2 and Table S2, Supplementary Materials). Among these, some explain how the amine network interacts with the phenolic tannin, such as the structure at 1666 Da (Figure S2, Supplementary Materials) in which two flavonoid monomers are linked to a hyperbranched tris(2-aminoethyl)amine-urea oligomer (structure **VII**) (Figure 9).

**Figure 10:**
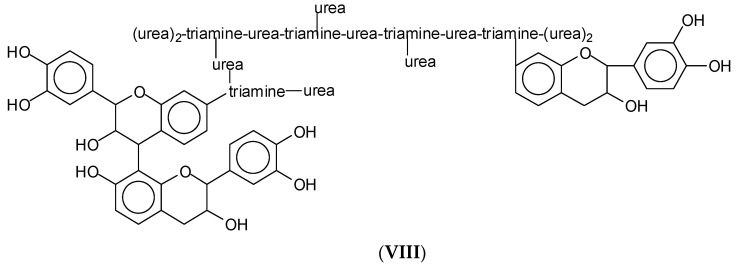
An example of a detected structure with three flavonoid units, one of which a flavonoid dimer linked to a triamine-urea hyperbrached network part.

Of equal interest is structure **VIII** (Figure 10), where a flavonoid dimer is also linked to the same hyperbranched amidoamine structure, indicating also that tannin oligomers, not only flavonoid monomers, link to the hyperbranched amidoamine structure.

Considering structures **VII** and **VIII,** the question arises with which groups of the hyperbranched structure do the flavonoid units of the tannin react. The substitution of the phenolic hydroxyl groups, which are quite acidic, by ammonia [20] and by amines is well known [21,22]. Thus, the substitution of some of the tannin phenolic -OHs by the free amino groups of tris(2-aminoethyl)amine is logically expected. The free primary amide -CONH_2_ groups of urea attached to the hyperbranched structure are, however, much less reactive.

**Figure 11:**
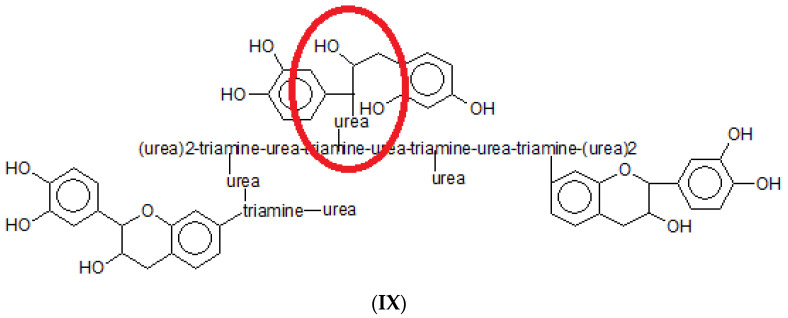
A rare example of a detected structure in which the amide group of a urea has reacted and linked to a flavoid unit of the tannin.

Urea is known to react with tannin but only on the opening of the heterocyclic flavonoid C-ring to stop tannin self-condensation [35], but not in general for substituting with an -NH- group the phenolic -OHs of flavonoids. This is so because the reactivity of the amide -CONH_2_ group is too low due to its far too low alkalinity for an acid–base reaction to occur with the mildly acidic phenolic -OHs. In tannin oligomers, it often occurs that there are a few flavonoid units where the C-ring has opened; some condensation with some urea terminal units of the triamine–urea hyperbranched structure can occur [35]. It is interesting to see by MALDI ToF that effectively a very limited amount of these primary amide groups are able to react with the tannin. This latter substitution reaction is not common, as over the full range of 3000 Da of the MALDI analysis, only one structure was found where the phenolic -OH of a flavonoid was substituted by the amide group of a terminal urea. Structure **IX** in Figure 11 is the one in question.

This is the case for this species as all the amino groups of the triamine were already linked either to urea or to flavonoids, and thus, the third flavonoid could not have reacted with anything else other than the free amide group of urea. It is thus evident the relative rarity of this linkage. It can occur, but to a limited level. 

**Figure 12:**
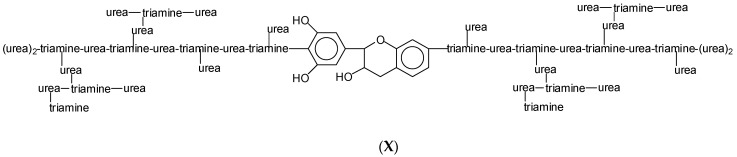
An example of a flavonoid unit linking two hyperbranched triamine-urea structures.

Species also occur showing two hyperbranched triamine–urea oligomers being linked by a tannin flavonoid unit, such as in Figure 12 the species at 3353 Da and 3369 Da (**X**) (Figure S2, Table S2 Supplementary Materials).

These species indicate that, when necessary, even the phenolic hydroxyl groups of the B-ring of the tannin can link to the hyperbranched triamine–urea oligomers.

From the above, it is also evident that the tannin oligomers do function as nucleation sites and hardeners of the tris(2-aminoethyl)amine-urea hyperbranched structure, yielding a better hardened adhesive network. It is also very worthwhile to consider from an environmental point of view that no formaldehyde and no aldehydes are used in such a wood adhesive system.

### 3.6. CP MAS ^13^C NMR

**Figure 13:**
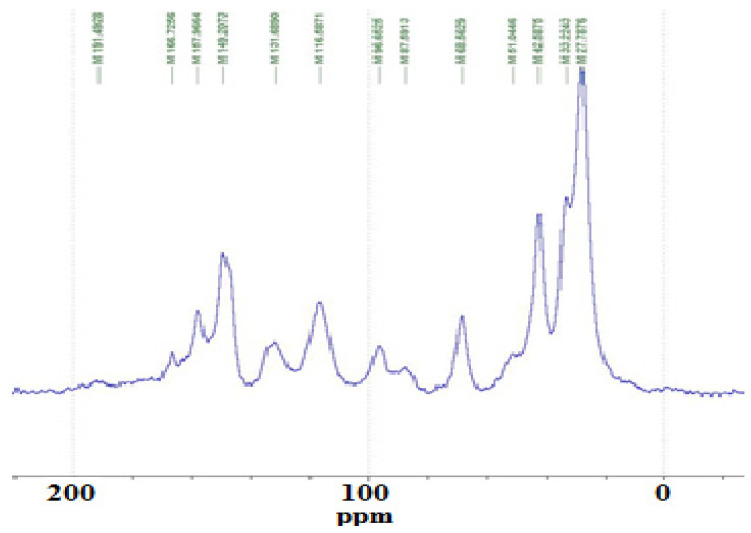
Cross-polarisation/magic-angle spinning (CP-MAS) ^13^C NMR spectrum of the reaction of the catechin model compound with hexamethylene diamine at 100 °C, NaOH-catalysed.

The shifts of the alpha carbons to the aliphatic diamine -NH groups should appear at a shift of 43–44 ppm if the diamine is reacted covalently with the tannin. The same alpha carbon for an unreacted aliphatic amine should appear at a calculated shift of between 41 and 42 ppm. Looking at the spectra of the 100 °C reaction in Figure 13 of the NaOH-catalysed catechin model with the amine, it can be noticed that the shift is at 42.6 ppm, indicating that at 100 °C, the amine reacted covalently. This is confirmed by other indications. The shift for the C in β to the covalently reacted amine should be at 30 ppm, while the unreacted one should be at 33–34 ppm. The first of these peaks is not visible, as covered by the 27–28 ppm wide peak, but it is assigned to the 33 ppm small shoulder. An indication that the type of linkages obtained in the 100 °C reaction is more uncertain, based on NMR evidence that ionic and covalent bonds may both be present, and is indicated by the relevant shift at 42.6 ppm. Previous research on the reaction of tannins and amines [21] had clearly implied that at higher temperatures such as 185 °C, the balance shifts prevalently towards amines covalently bonding with the flavonoid. Moreover, the markedly lower 157 ppm peak further indicates and confirms clearly that covalent bonds between the catechin -OH groups and the amine have formed. As this shift refers to the C5 and C7 carbons carrying the -OH groups on the catechin A-ring, its marked decrease shows that their proportion is lower as they reacted with the amine. This is confirmed by the appearance of the small double peak at 135 ppm attached to the 131 ppm peak characteristic of the C1 of unreacted catechin. The 135 ppm peak is assigned to the C5 and C7 that reacted covalently with an amine, indicating that there was a considerable substitution reaction of the -OHs of the flavonoid A-ring. The alcoholic character -OH on the C3 site appears to have not reacted, or to have reacted very little, covalently with the amine as shown by the catechin C3 peak at the unaltered 68–72 ppm shift for the 100 °C reaction. This is in contrast to what was previously observed at higher temperatures of reaction (185 °C) where this peak disappeared completely, indicating that the reaction of the amine on this site did occur at the higher temperature [21]. Again, this must be kept in mind when pressing particleboard at 180 °C because, while the board faces reach effectively 180 °C, the board core only reaches between 115 °C and 120 °C [24,25]. This indicates that there is a difference in the chemical structure of this adhesive in the board core and its surfaces.

From Figure 5 of the catechin plus amine reaction, the phenolic -OHs that react preferentially at 100 °C appear to be predominantly on the flavonoid A-ring, first, as shown by the shoulder at 135 ppm on the side of the 131 ppm peak and as the 149 ppm peak belonging to the aromatic B-ring carbons carrying the phenolic -OH groups is not markedly smaller than in unreacted catechin. This is contrary to what was found at higher temperatures (185 °C) where all phenolic -OHs on both the A and B-rings reacted [21]. Again, this is a difference to keep in mind about the structure of the hardened resins in a cured wood particleboard.

**Figure 14:**
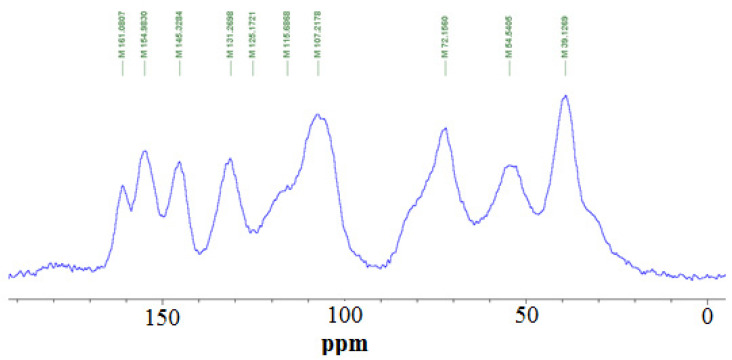
Cross-polarisation/magic-angle spinning (CP-MAS) ^13^C NMR spectrum of the reaction product of mimosa flavonoid tannin with the hyperbranched tris(2-amino ethyl)amine-urea polymer at 100 °C.

Figure 14 shows the CP MAS ^13^C NMR spectrum of the adhesive obtained by reacting mimosa tannin with the preprepared hyperbranched tris(2-aminoethyl)amine-urea. The spectrum is mainly dominated by the tannins’ signals, although the amine and urea signals are also visible. The 161 ppm peak is assigned to the C=O carbonyl of urea [36]. The fact that it shifted to 161 ppm from 166 ppm in Figure 5 indicates that in the case of the reaction with the tannin, even the amide groups of urea could react by substituting the phenolic or alcoholic -OH groups of the tannin. The peak at 145 ppm is assigned to the C3′ and C4′ of the tannin, while the 154 ppm peak is assigned to the tannin aromatic C-OH on C7 [16,37]. The lower intensity of this peak in relation to the ^13^C NMR spectra of mimosa tannin alone indicates that some of the phenolic -OH groups on the A-rings reacted with the amine and transformed into -NH- groups. This is supported by the peak at 131 ppm that is clearly formed in Figure 6 by two peak points. The first at 131 ppm is the C1′ of the flavonoid [16,37]. The equally high second point at 136 ppm is the signal of the aromatic carbons of the flavonoid in which the phenolic -OHs of both the A and B rings have been substituted by -NH- groups. This confirms that the tannin and the hyperbranched tris(2-aminoethyl)amine-urea polymer are covalently linked. The very wide shoulder at 135 ppm is formed by the superposition of the signals of C2′, C5′, and C6′ [16,37,38]. The wide peak centered at 107 ppm is assigned to the superposition of the C4-C8 and C4-C6 interflavonoid links between the tannin flavonoid units [16,37,38]. The small peak of the tannin flavonoid C10 is assigned to the small shoulder at 101 ppm. The peak at 72 ppm is assigned to C3, and the wide shoulder at 78+ ppm is assigned to the tannin C2 [16,37,38]. The peak at 54 ppm is clearly composed of two signals. The 54 ppm shift is that of the carbons directly linked to the tertiary N of the tris(2-aminoethyl)amine, while the strong peak at 39 ppm is assigned to the carbons attached to -NH_2_ of the tris(2-aminoethyl)amine. The second shift attached at 54 ppm is the signal of the reacted flavonoid C4 site, indicating that the flavonoids are oligomer chains, a fact confirmed by the existence of the C4-C8 and C4-C6 interflavonoid link shifts at 107 ppm [16,37,38]. The shoulder at 32 ppm is assigned to the tannin’s unreacted C4, thus at the end of the chain. In the case that the -NH- groups have substituted and also some of the alcoholic character -OHs on the tannin C3, these would also appear at 39 ppm, but this is superposed by the strong tris(2-aminoethyl)amine peat at 39 ppm.

To conclude, the NMR in Figure 6 confirms that tannin oligomers are linked covalently to the hyperbranched tris(2-aminoethyl)amine-urea. The amine -NH- group appears to be the one having prevalently substituted some of the phenolic -OHs, both on the A-ring and even on the B-ring. This confirms previous higher temperature results [21]. The substitution of the alcoholic character -OH on the tannin C3 is much less, or much less likely, but doubtful.

## 4. Conclusions

Non-aldehyde resins made by tannin copolymerized with a hyperbranched network of tris(2-aminoethyl)amine-urea have been shown to yield good thermosetting adhesives for wood particleboard, satisfying the requirements of relevant standards for the internal bond (IB) strength. The lack of aldehyde and the abundance of still-free amine and amide functions help to reduce to emissions to 0.0, even the little formaldehyde emissions that could have been generated by the heating of wood. Tannin allows for the curing of the triamine–urea network at the relatively low press temperature of 180 °C for today’s particleboard manufacturing. This indicates that the resin is also able to cure at the lower temperatures of 115–120 °C prevalent in the board core. The chemical analysis, in particular by MALDI ToF and ^13^C NMR, showed that the majority of the cross-linking reactions were that of the substitution of the tannin hydroxyls by the amino groups of the triamine. The reaction of the tannin with the still-free amide groups of urea is rare, but it might occur with the rarer tannin flavonoid units in which the heterocyclic ring has opened. Due to the temperature gradient between the particleboard surfaces and core during hot pressing, there is likely to be a difference in the cured resin structure in the surfaces and the board core.

## Figures and Tables

**Table 1:** Weight proportions of HBP:tannin solids in the resin tested.

Resin	Proportions	pH	Resin Name
HBP:Tannin	0:1 (Control)	10	T0
HBP:Tannin	1:4	10	T1
HBP:Tannin	1:5	10	T2
HBP:Tannin	1:6	10	T3

**Table 2:** Viscosity variation for the four formulations tested.

Resin	Rotating Rate (r/s)	Viscosity (mPa.s)
T0	1	6900–7000
T1	1	4600–4800
T2	1	3200–3300
T3	1	4400–4500
T0	2.5	3880–3920
T1	2.5	2680–2720
T2	2.5	1840–1880
T3	2.5	2440–2480
T0	5	2660–2700
T1	5	1780–1800
T2	5	1260–1280
T3	5	1600–1620
T0	10	1870–1890
T1	10	1240–1250
T2	10	900–910
T3	10	1100–1110
T0	20	1365–1375
T1	20	905–910
T2	20	670–575
T3	20	795–805

**Table 3:** Average results of tests of particleboard panels bonded with T0, T1, T2, and T3 resins.

Resin	Solid Content (%)	Bending Strength (MPa)	Internal Bond Strength (MPa)	Board Density (g/cm^3^)	2 h Thickness Swelling (%)	24 h Thickness Swelling (%)
T0	41.0 ± 0.43	9.08 ± 1.83	0.24 ± 0.04	0.810 ± 0.02	62.1 ± 4.2	63.5 ± 2.1
T1	40.3 ± 0.46	11.53 ± 2.11	0.62 ± 0.11	0.770 ± 0.03	36.6 ± 1.4	51.6 ± 1.0
T2	39.8 ± 0.32	11.22 ± 1.12	0.64 ± 0.12	0.770 ± 0.01	36.1 ± 1.9	49.4 ± 1.5
T3	39.5 ± 0.10	13.48 ± 1.32	0.70 ± 0.07	0.750 ± 0.01	36.7 ± 1.5	50.2 ± 2.7
EN312			≥0.35			

## Data Availability

Not applicable.

## Supplementary Materials

The following supporting information can be downloaded at: https://www.mdpi.com/article/10.3390/polym15040890/s1, Figure S1: MALDI ToF spectra of hyperbranched tris(2-aminoethyl) amine-urea oligomers; Table S1: Structure assignment for the peaks of the hyperbranched triamine–urea reaction products; Figure S2: MALDI ToF spectra of tannin units linked to hyperbranched tris(2-aminoethyl) amine-urea oligomers; Table S2: Structure assignment for the peaks of the reaction tannin-hyperbranched triamine–urea resin T2. Figure S3: CP MAS ^13^C NMR spectrum of mimosa condensed tannin.
