# Supplementary material for: Highly Branched Tannin-Tris(2-aminoethyl)amine-Urea Wood Adhesives
Source: Polymers (Basel). 2023 Feb 10;15(4):890. doi: 10.3390/polym15040890 (PMC9960572; doi:10.3390/polym15040890)
Supplement: Supplementary file 1 [file polymers-15-00890-s001.zip › polymers-2211299-supplementary.pdf]

SUPPLEMENTARY MATERIAL

Wood Adhesives by Tannin Copolymerization with Hyperbranched Tris(2aminoethyl)amine-Urea Networks

B.Zhang, X.Chen, A.Pizzi*, M.Petrissans, S.Dumarcay, A.Petrissans, X.Zhou, G.Du, B.Colin, X.Xi

MALDI KEY TRISAMINE+UREA ALONE AND TRISAMINE+UREA+TANNIN (T2)

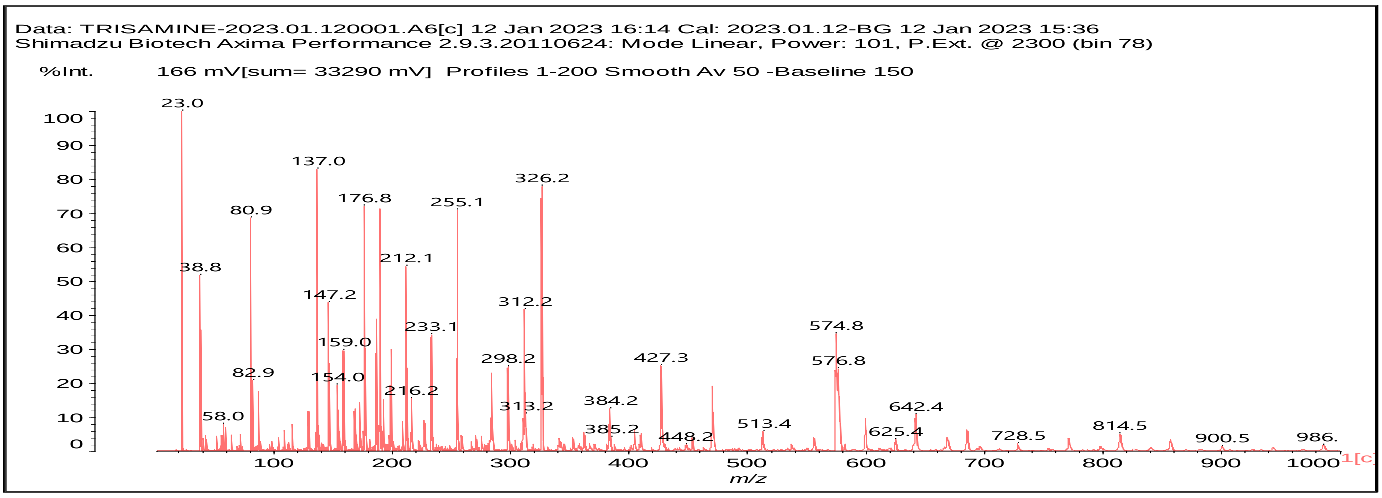

A

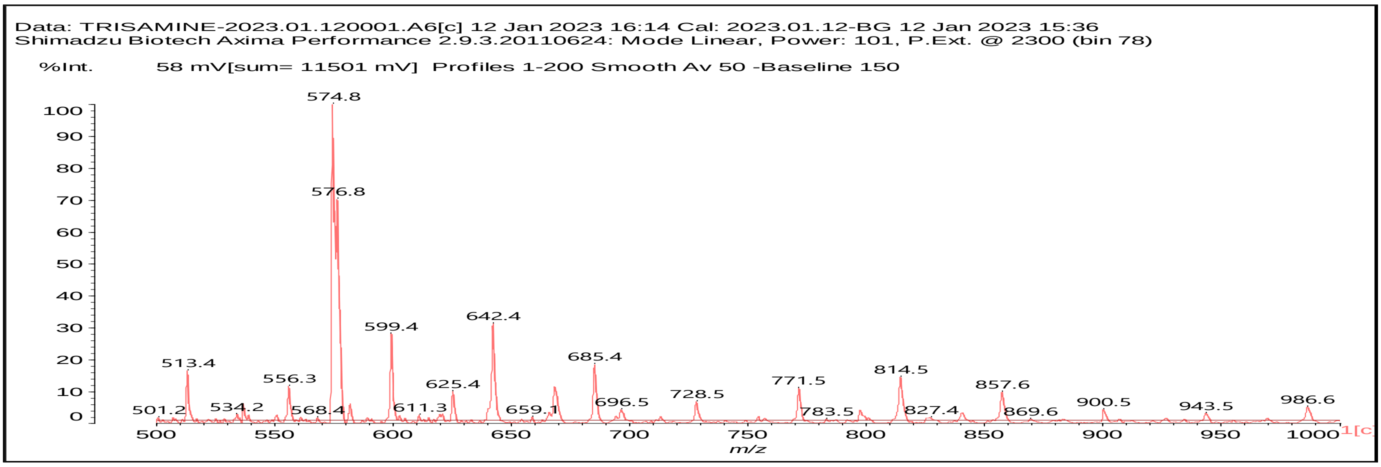

B

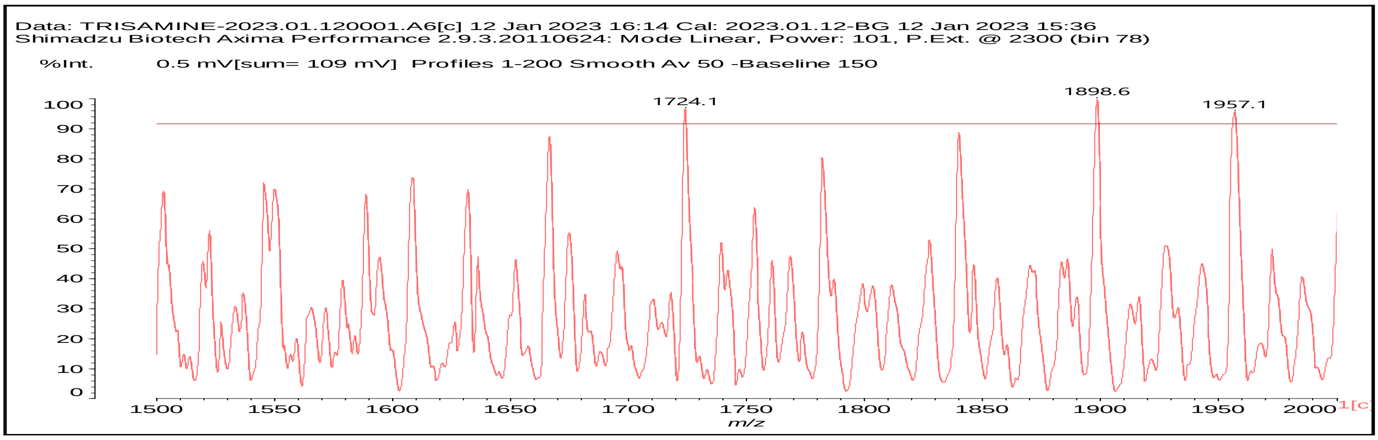

C

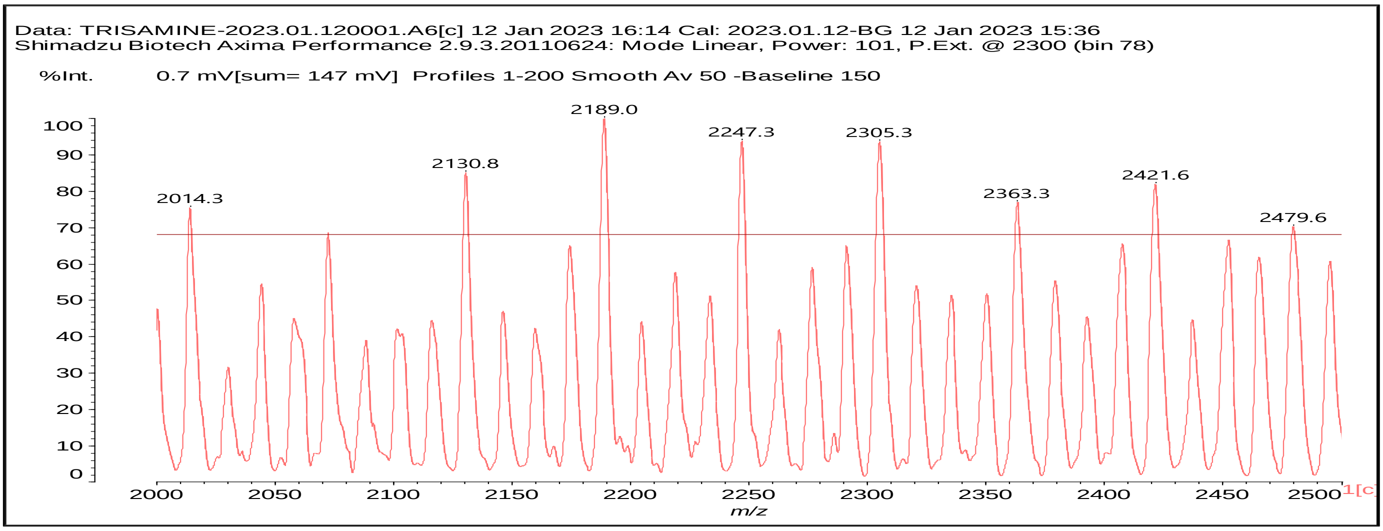

D

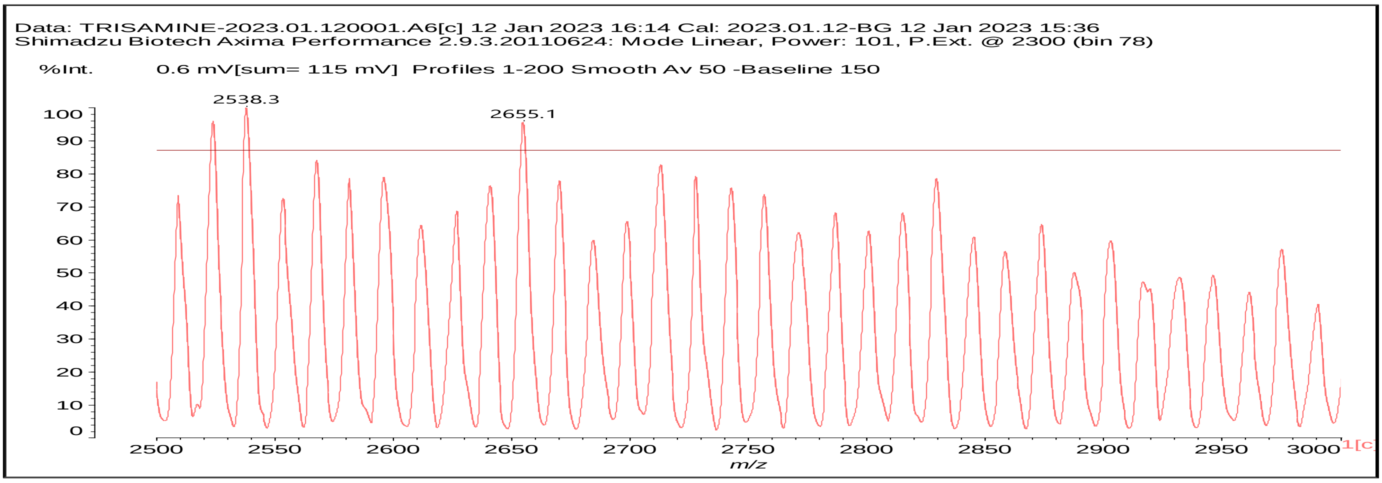

E

Figure S1 , A,B, C, D, E. MALDI ToF Spectra of hyperbranched tris(2-aminoethyl) amine-urea oligomers

Table S1. Structure assignement for the peaks of the hyperbranched trisamine-urea reaction products.

The peaks with "*" exist also in the triamine+urea+tannin cases

58 Da = urea, deprotonated, no Na+

83 Da = urea, with Na+

147 Da = Tris(2-aminoethyl)amine, no Na+

*212 Da = with Na+, tris(2-aminomethyl)amine-urea.

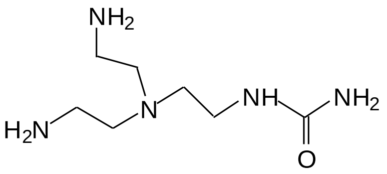

216 Da =no Na+, protonated, calc. 216 Da

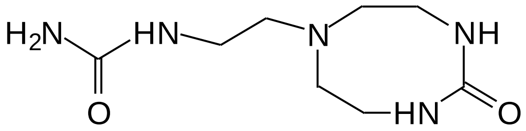

*232 Da = no Na+, **255 Da** with Na+, of +, tris(2-aminomethyl)amine-(urea)$_2$.

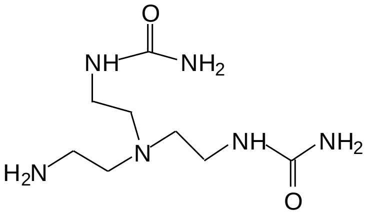

*298 Da = with Na+, triamine-(urea)$_3$

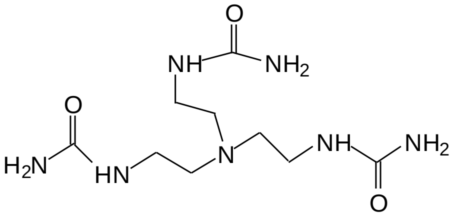

384 Da = with Na+, triamine-urea-triamine-urea

*427 Da = with Na+, urea-triamine-urea-triamine-urea

*513 Da = with Na+, triamine-urea-triamine-urea-triamine **OR**

*513 Da =no Na+, (urea)$_2$-triamine-urea-triamine-(urea)$_2$

534 Da = no Na+, triamine-urea-triamine-urea-triamine-urea

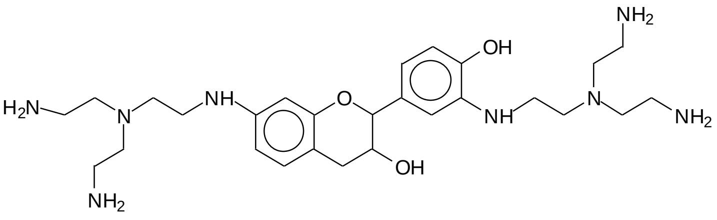

556 Da = with Na+, triamine-urea-triamine-urea-triamine-urea

*575-577 Da = no Na+, urea-triamine-urea-triamine-urea-triamine-urea **OR**

*642 Da = with Na+, (urea)$_2$-triamine-urea-triamine-urea-triamine-urea

659 Da = no Na+, (urea)$_2$-triamine-urea-triamine-urea-triamine-(urea)$_2$

728 Da = with Na+, urea-triamine-urea-triamine-urea-triamine-urea-triamine

*771 Da = with Na+, (urea)$_2$-triamine-urea-triamine-urea-triamine-urea-triamine

*814 Da = with Na+, (urea)$_2$-triamine-urea-triamine-urea-triamine-urea-triamine-urea

986 Da = with Na+, (urea)$_2$-triamine(urea)-urea-triamine(urea)-urea-triamine(urea)-urea-triamine-(urea)$_2$

*1115 Da =with Na+,

```
                         urea
                         |
(urea)2-triamine-urea-triamine-urea-triamine-urea-triamine-(urea)2
           |                             |
           urea                          urea
            |
            triamine
```

1158 Da , with Na+,

```
                         urea
                         |
(urea)2-triamine-urea-triamine-urea-triamine-urea-triamine-(urea)2
           |                             |
           urea                          urea
            |
            triamine—urea
```

1244 Da =with Na+,

```
                           triamine
                           |
                         urea
                         |
(urea)2-triamine-urea-triamine-urea-triamine-urea-triamine-(urea)2
           |                             |
           urea                          urea
            |
            triamine
```

OR

1287 Da = Na+,

```
                           triamine——
                           |
                         urea
                         |
(urea)2-triamine-urea-triamine-urea-triamine-urea-triamine-(urea)2
           |                             |
           urea                          urea
            |
            triamine—urea
```

1331 Da = with Na+,

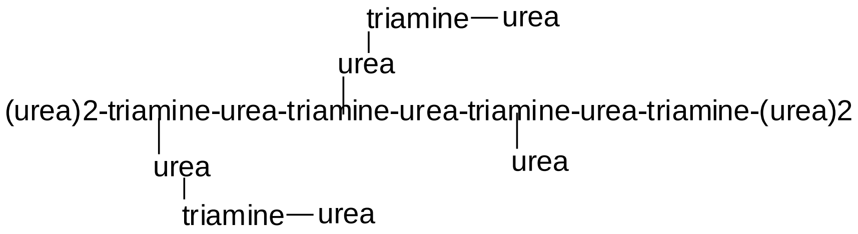

*1374 Da = wih Na+

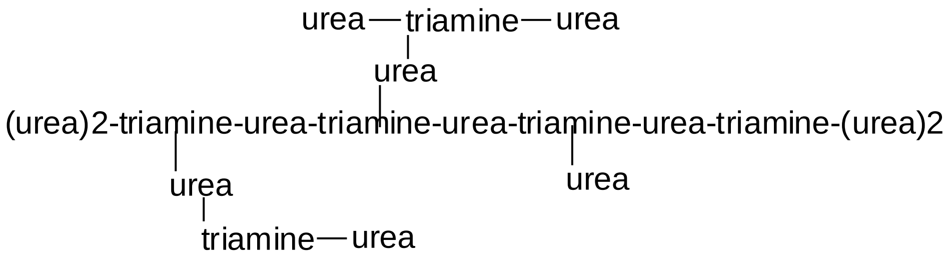

1417 Da = with Na+,

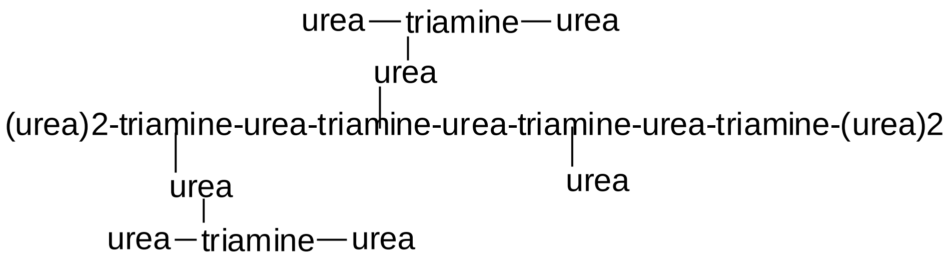

1546 Da = with Na+

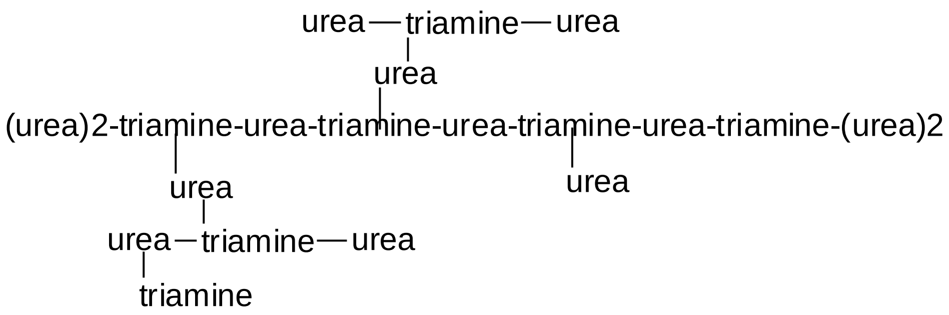

1724 to 1898 Da, difference of 172 -174 Da = -triamine-urea, repeating motive

1957-2130 – 2305-2479-2655 Da series just adds a series of = -triamine-urea, repeating motive

*1957 Da = with Na+

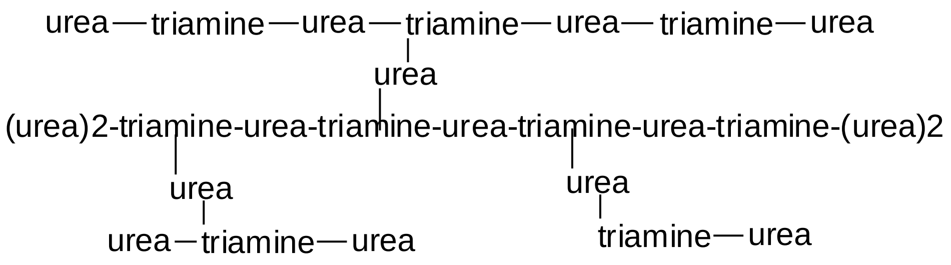

*2130 Da= with Na+

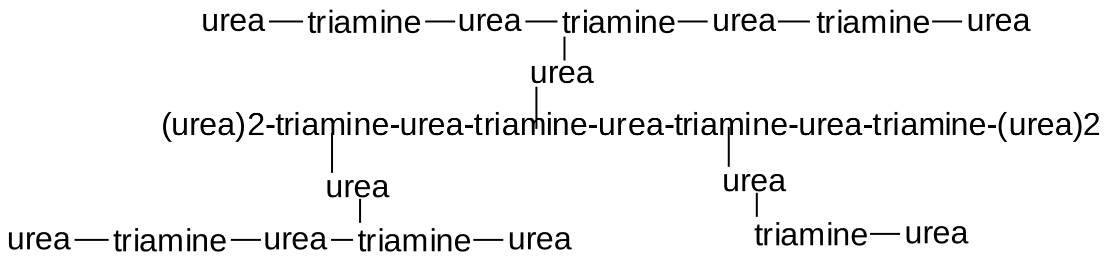

*2305 Da =with Na+

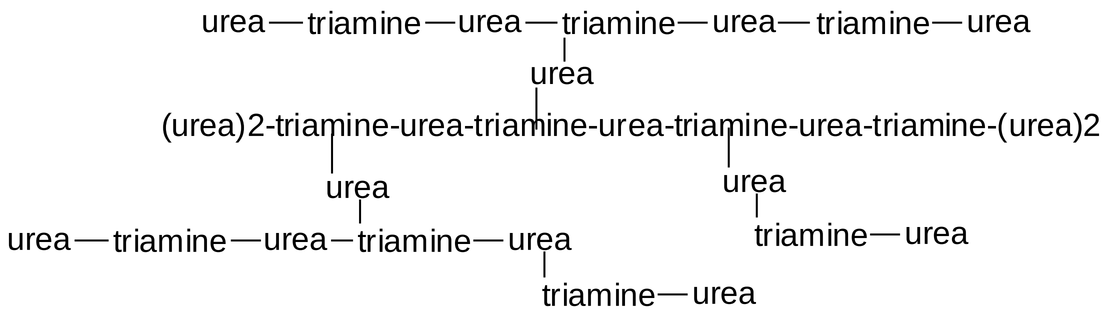

*2479 Da = with Na+

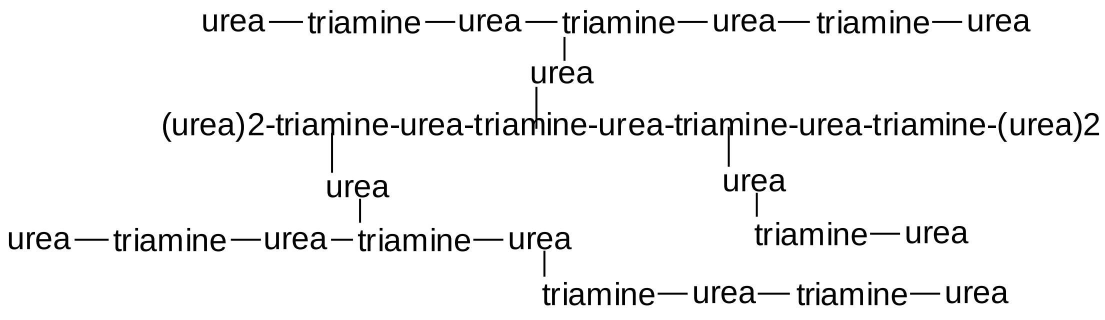

2655 Da =with Na+

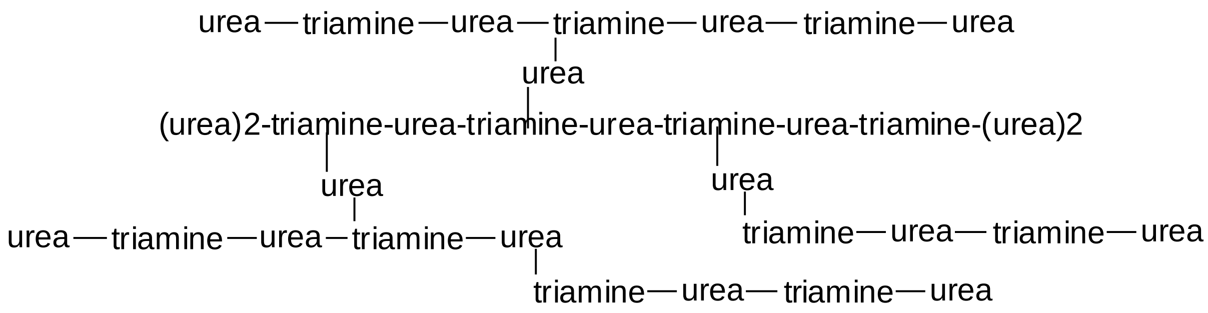

Table S2. Structure assignement for the peaks of the reaction tannin-hyperbranched trisamine-urea resin T2. To note that in Table A1 are shown by an "*" all the peaks that are also present in the T2 spectra but that have not reacted with tannin units.

276 Da = no Na+, fisetinidin, protonated

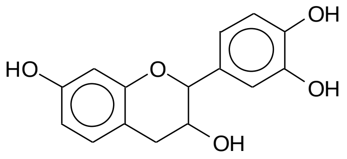

306 Da = no Na+, gallocatechin

330 Da = with Na+, gallocatechin,

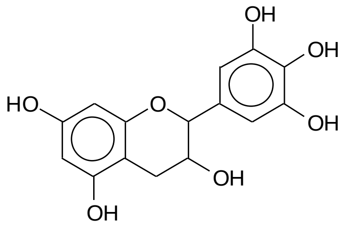

405 Da = no Na+, triamine-fisetinidin (T2), protonated calc. 404 Da.

*427 Da = with Na+, urea-triamine-urea-triamine-urea

OR with Na+, triamine-fisetinidin (T2)

470 Da = with Na+, triamine-urea-fisetinidin (T2) OK, protonated, calc. 469 Da

551 Da = with Na+, OK, deprotonated, calc; 552 Da

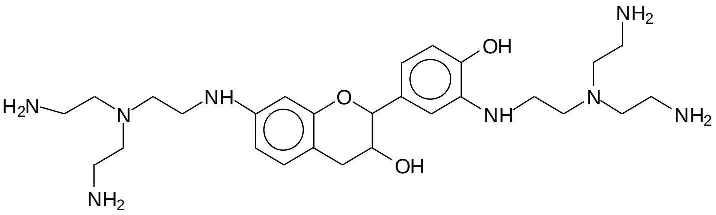

574 Da =no Na+, triamine-urea-triamine-fisetinidin (T2)

599 Da = with Na+, same as 574, triamine-urea-triamine-Fisetinidin (T2)

657 Da = with Na+, triamine-urea-triamine-urea-robinetinidin (T2)

OR

657 Da = no Na+, showing triamine alone linking two fisetinidin flavonoids

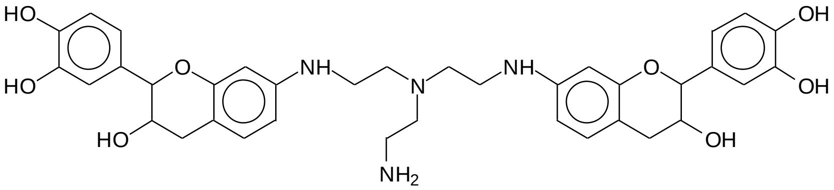

685 Da = with Na+, urea-triamine-urea-triamine-urea-fisetinidin (T2)

699 Da = with Na+, urea-triamine-urea-triamine-urea-robinetinidin (T2)

728 Da = with Na+, (urea)$_2$-triamine-urea-triamine-urea-fisetinidinn But also present without flavonoid

771 Da = with Na+, (urea)$_2$-triamine-urea-triamine-(urea)$_2$-fisetinidin. But also present without flavonoid

945 Da = no Na+, protonated, calc. 946 Da

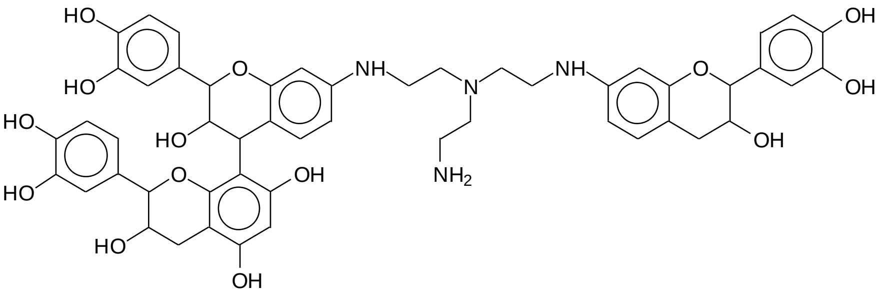

OR/AND

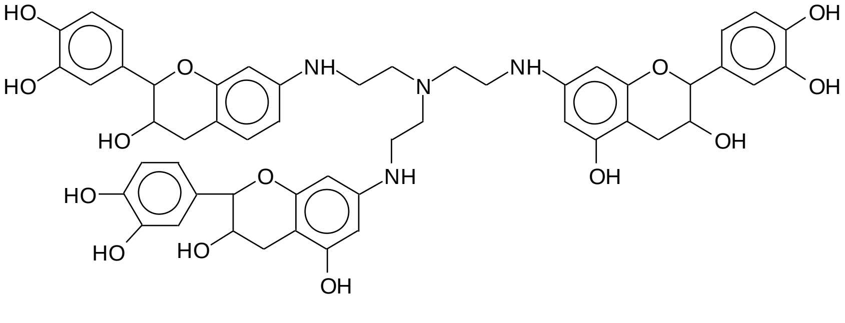

1242 Da = with Na+, **fisetinidin**-(urea)$_2$-triamine(urea)-urea-triamine(urea)-urea-triamine(urea)-urea-triamine-(urea)$_2$

1435 Da = with Na+, fisetinidin+1158 Da, deprotonated, calc 1436 Da

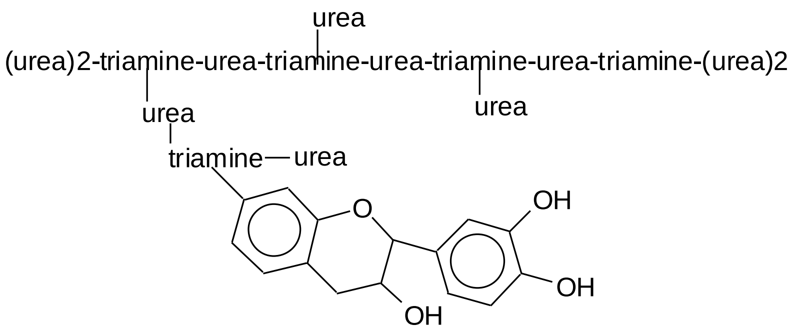

1666 Da = no Na+ , (fisetinidin)$_2$+1158 Da, deprtonated, calc. 1667 Da

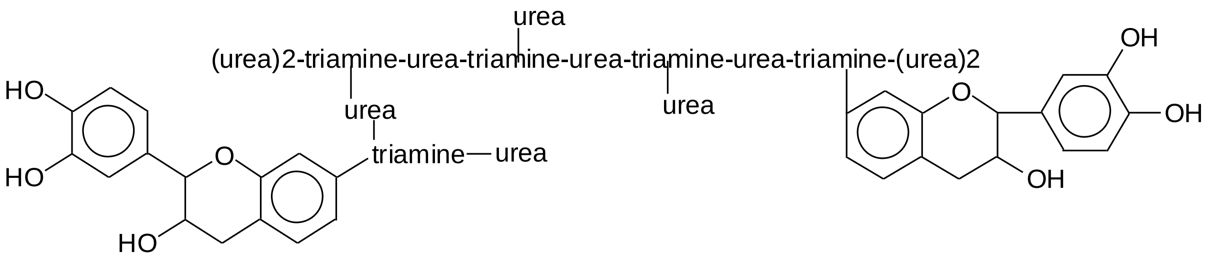

1938 Da = no Na+

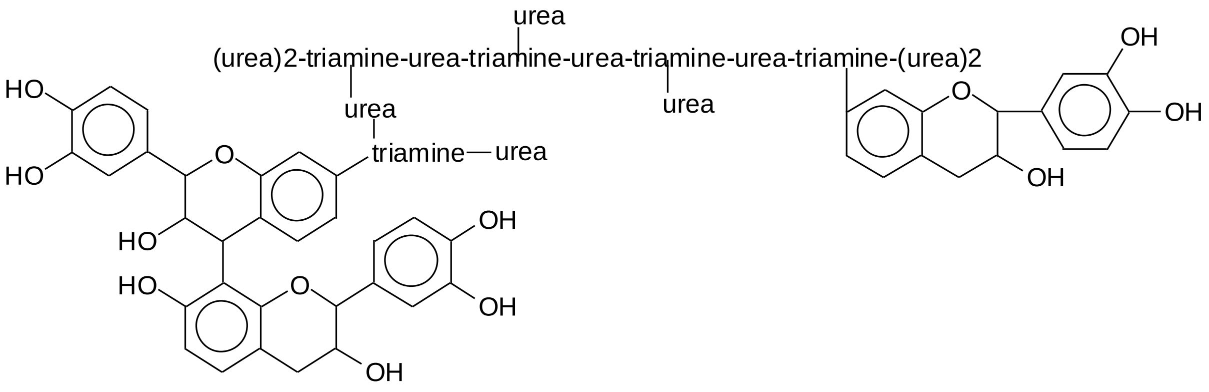

1945 Da = with Na+, fisetinidin linked to urea+ 1666 Da. IMPORTANT, the Flavonoid Links also to the urea, an amide. As it cannot do otherwise in this set-up

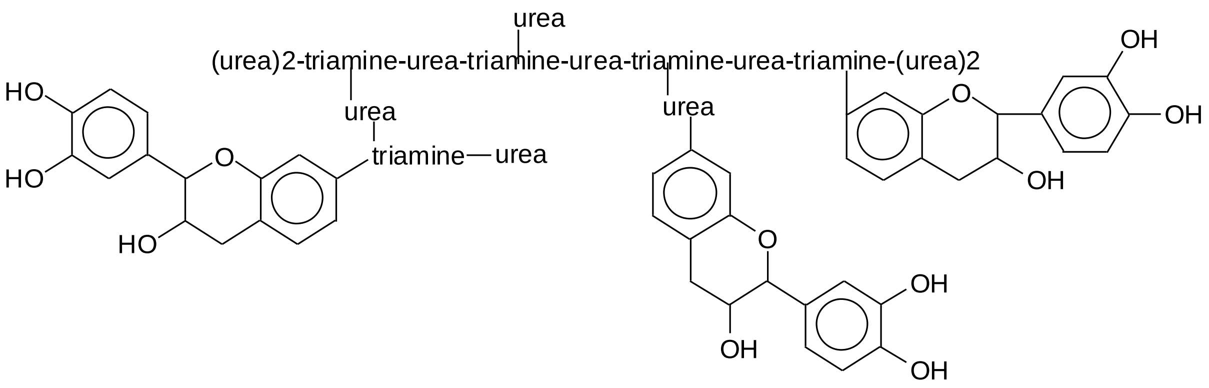

1956 Da =no Na+, protonated , calc 1955 Da

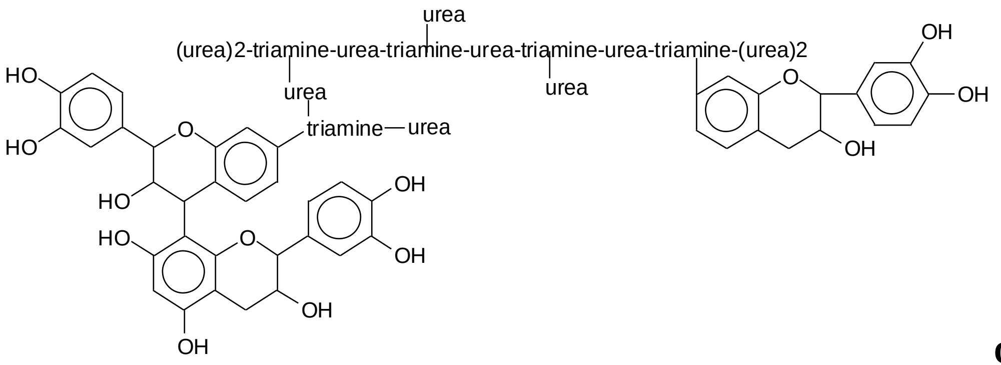

OR

*1957 Da = with Na+

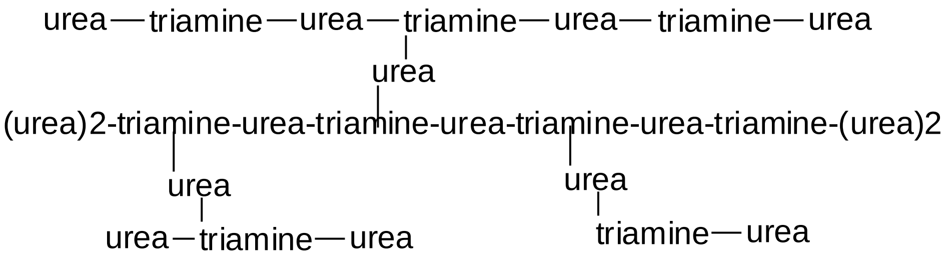

2538 Da = no Na+, fisetinidin+2305

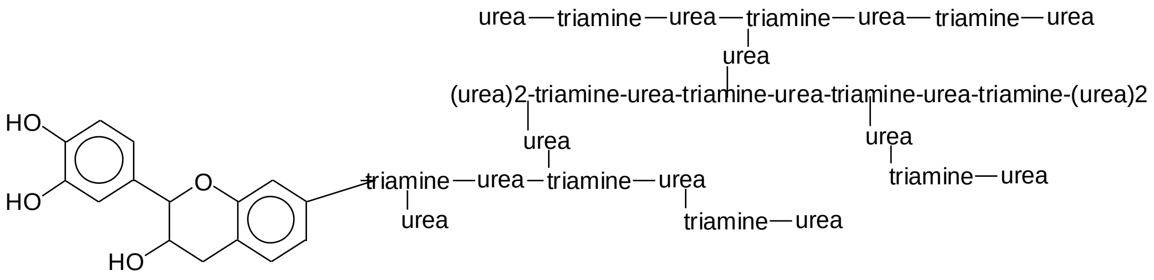

2829 Da =no Na+, 2538 +1xgallocatechin

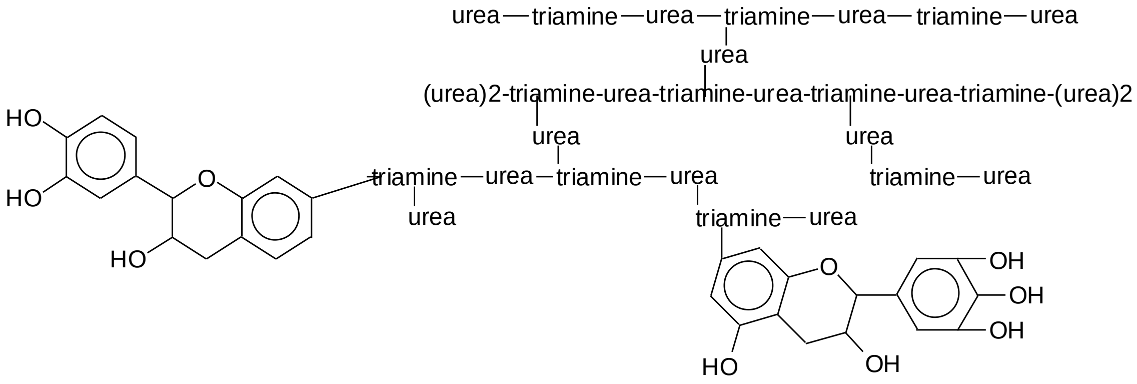

2903 Da = no Na+, 2655 +robinetinidin, protonated OK

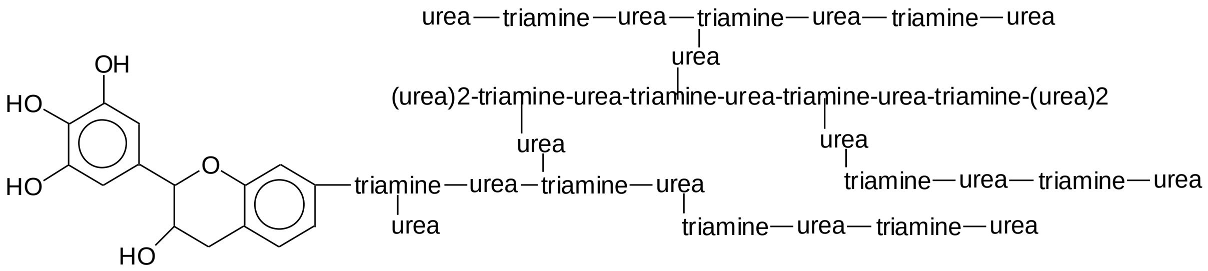

2917 Da = no Na+, +1xgallocatechin, deprotonatedOK

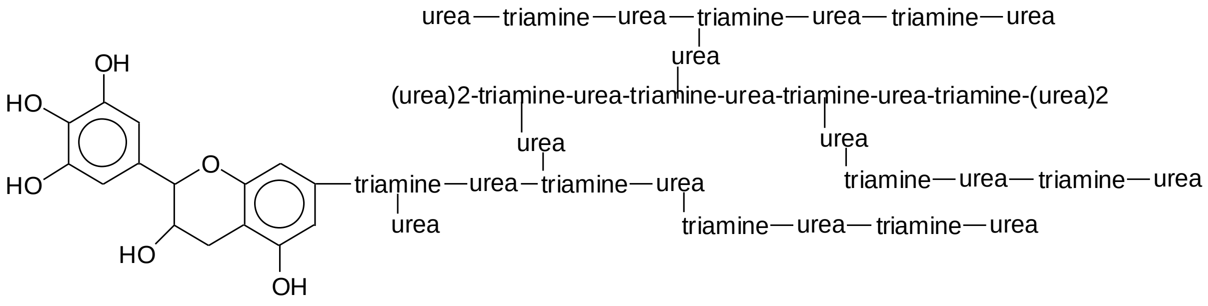

3353 Da =with Na+, two 1546 Da triamine-urea oligomers linked by a tannin fisestinidin unit

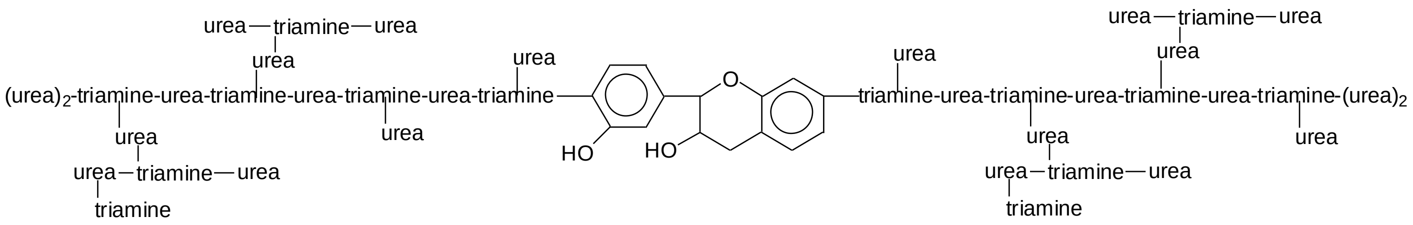

3369 Da = with Na+, +, two 1546 Da triamine-urea oligomers linked by a tannin robinetinidin unit

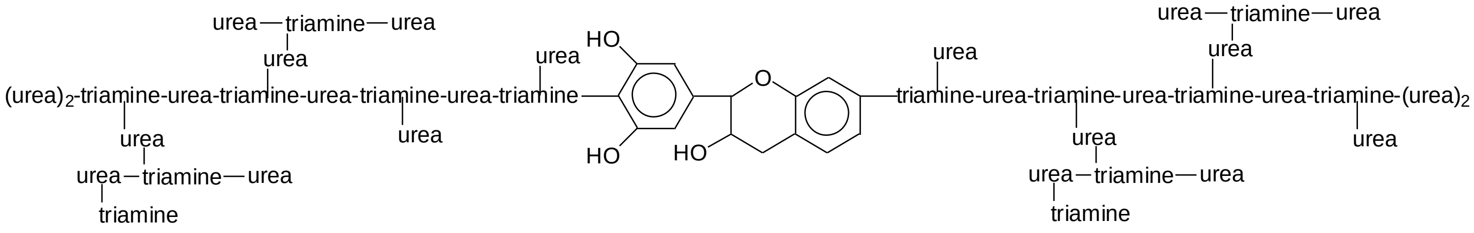

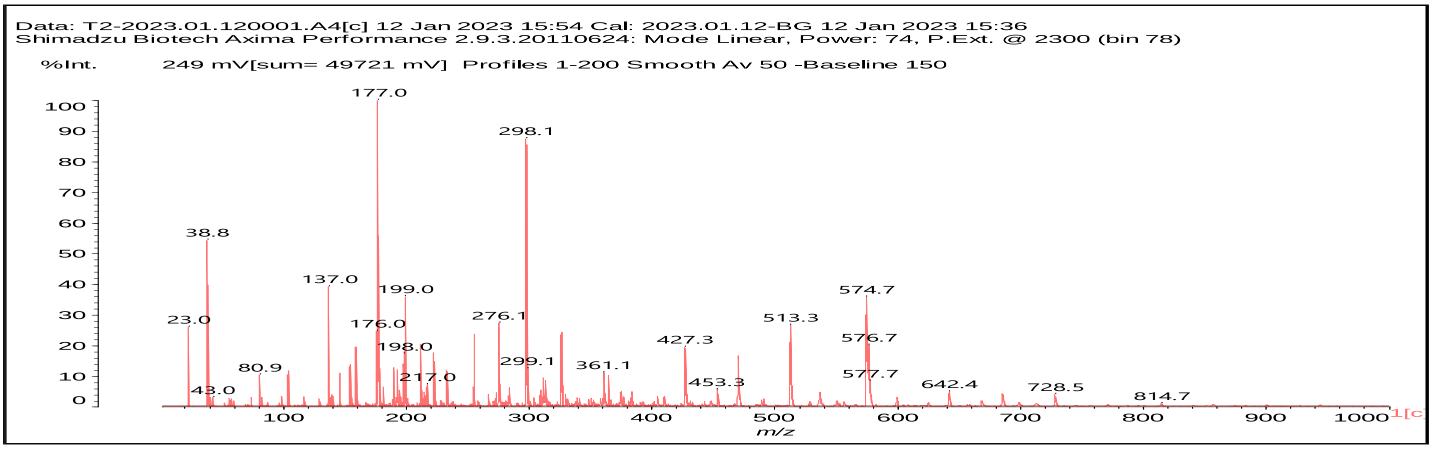
Data: T2-2023.01.120001.A4[c] 12 Jan 2023 15:54 Cal: 2023.01.12-BG 12 Jan 2023 15:36
Shimadzu Biotech Axima Performance 2.9.3.20110624: Mode Linear, Power: 74, P.Ext. @ 2300 (bin 78)
%Int.
249 mV[sum= 49721 mV] Profiles 1-200 Smooth Av 50 -Baseline 150
177.0
298.1
38.8
137.0
199.0
574.7
23.0
176.0
276.1
513.3
576.7
198.0
299.1
427.3
361.1
80.9
217.0
577.7
453.3
642.4
728.5
43.0
814.7
m/z

A

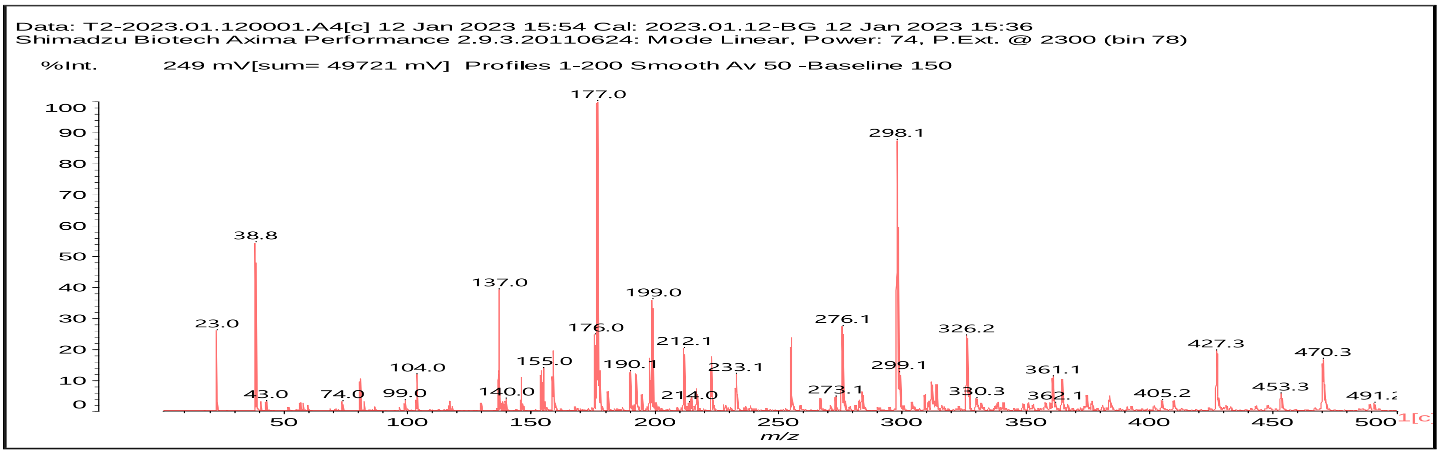
Data: T2-2023.01.120001.A4[c] 12 Jan 2023 15:54 Cal: 2023.01.12-BG 12 Jan 2023 15:36
Shimadzu Biotech Axima Performance 2.9.3.20110624: Mode Linear, Power: 74, P.Ext. @ 2300 (bin 78)
%Int.
249 mV[sum= 49721 mV] Profiles 1-200 Smooth Av 50 -Baseline 150
177.0
298.1
38.8
137.0
199.0
23.0
176.0
276.1
326.2
212.1
427.3
470.3
104.0
155.0
190.1
233.1
299.1
361.1
453.3
43.0
74.0
99.0
140.0
214.0
273.1
330.3
362.1
405.2
m/z

B

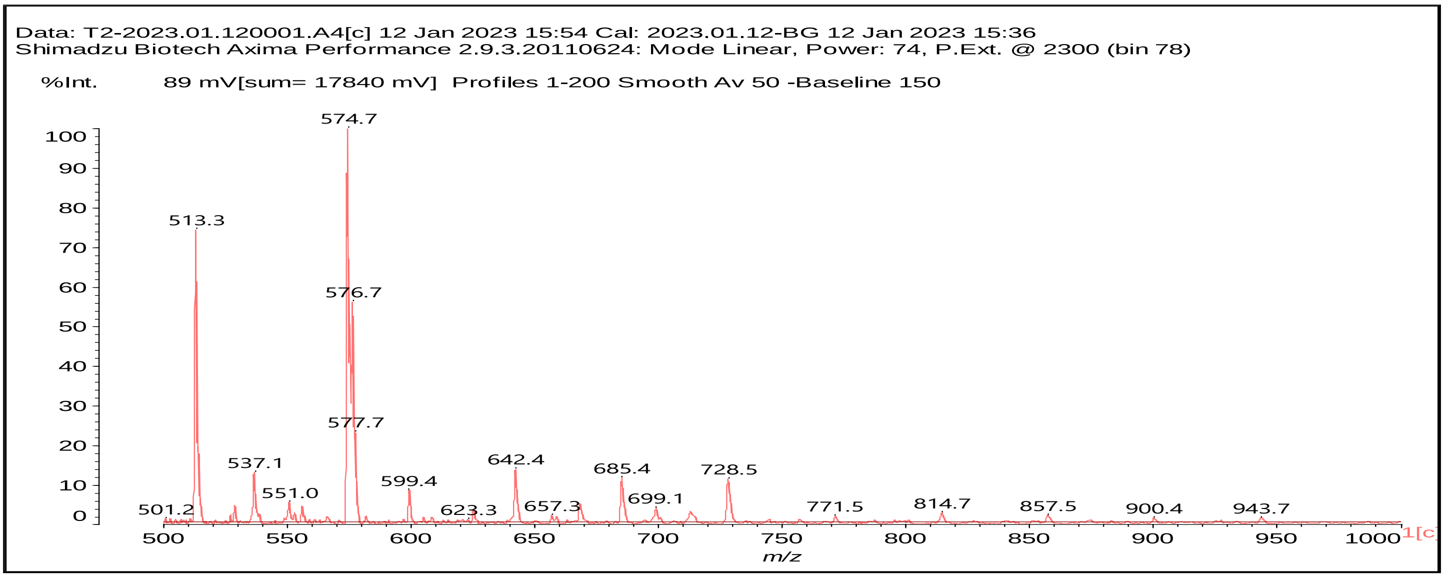
Data: T2-2023.01.120001.A4[c] 12 Jan 2023 15:54 Cal: 2023.01.12-BG 12 Jan 2023 15:36
Shimadzu Biotech Axima Performance 2.9.3.20110624: Mode Linear, Power: 74, P.Ext. @ 2300 (bin 78)
%Int.
89 mV[sum= 17840 mV] Profiles 1-200 Smooth Av 50 -Baseline 150
574.7
513.3
576.7
577.7
537.1
642.4
685.4
728.5
551.0
599.4
699.1
501.2
623.3
657.3
771.5
814.7
857.5
900.4
943.7
m/z

C

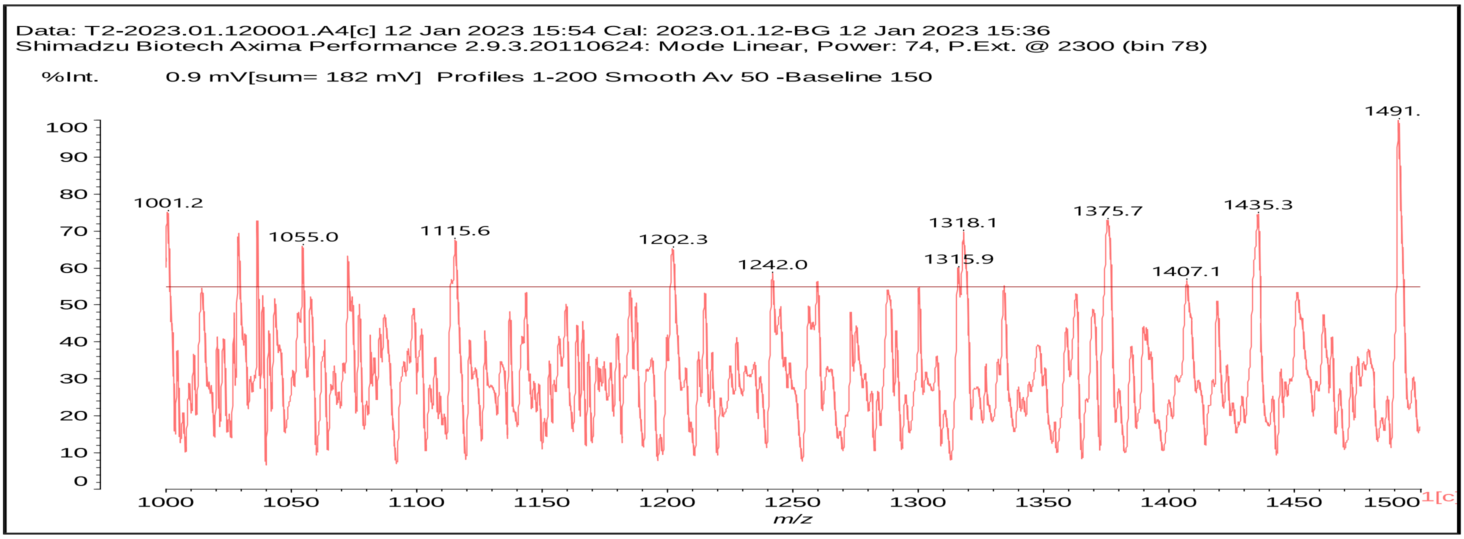
Data: T2-2023.01.120001.A4[c] 12 Jan 2023 15:54 Cal: 2023.01.12-BG 12 Jan 2023 15:36
Shimadzu Biotech Axima Performance 2.9.3.20110624: Mode Linear, Power: 74, P.Ext. @ 2300 (bin 78)
%Int. 0.9 mV[sum= 182 mV] Profiles 1-200 Smooth Av 50 -Baseline 150
m/z

D

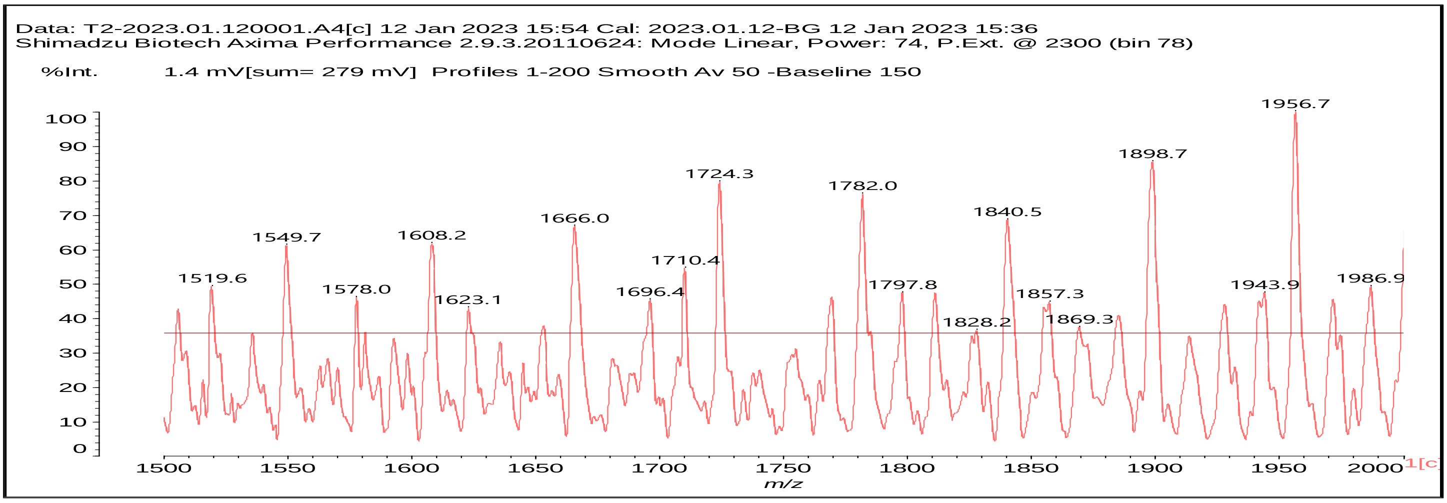
Data: T2-2023.01.120001.A4[c] 12 Jan 2023 15:54 Cal: 2023.01.12-BG 12 Jan 2023 15:36
Shimadzu Biotech Axima Performance 2.9.3.20110624: Mode Linear, Power: 74, P.Ext. @ 2300 (bin 78)
%Int. 1.4 mV[sum= 279 mV] Profiles 1-200 Smooth Av 50 -Baseline 150
m/z

E

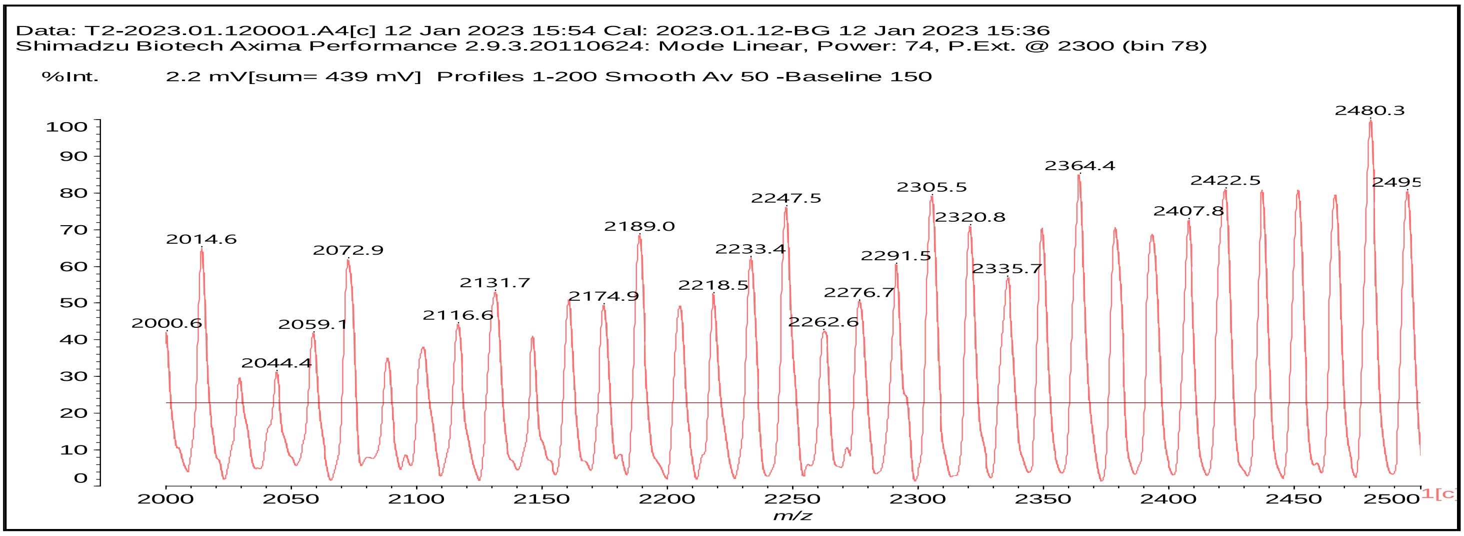
Data: T2-2023.01.120001.A4[c] 12 Jan 2023 15:54 Cal: 2023.01.12-BG 12 Jan 2023 15:36
Shimadzu Biotech Axima Performance 2.9.3.20110624: Mode Linear, Power: 74, P.Ext. @ 2300 (bin 78)
%Int. 2.2 mV[sum= 439 mV] Profiles 1-200 Smooth Av 50 -Baseline 150
m/z

F

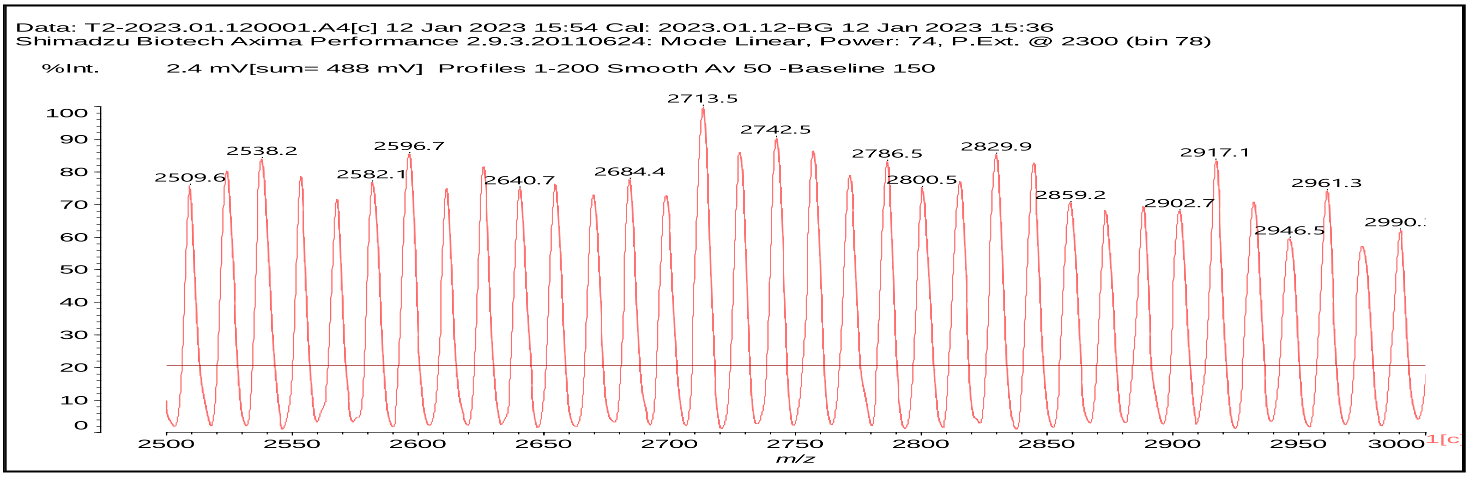

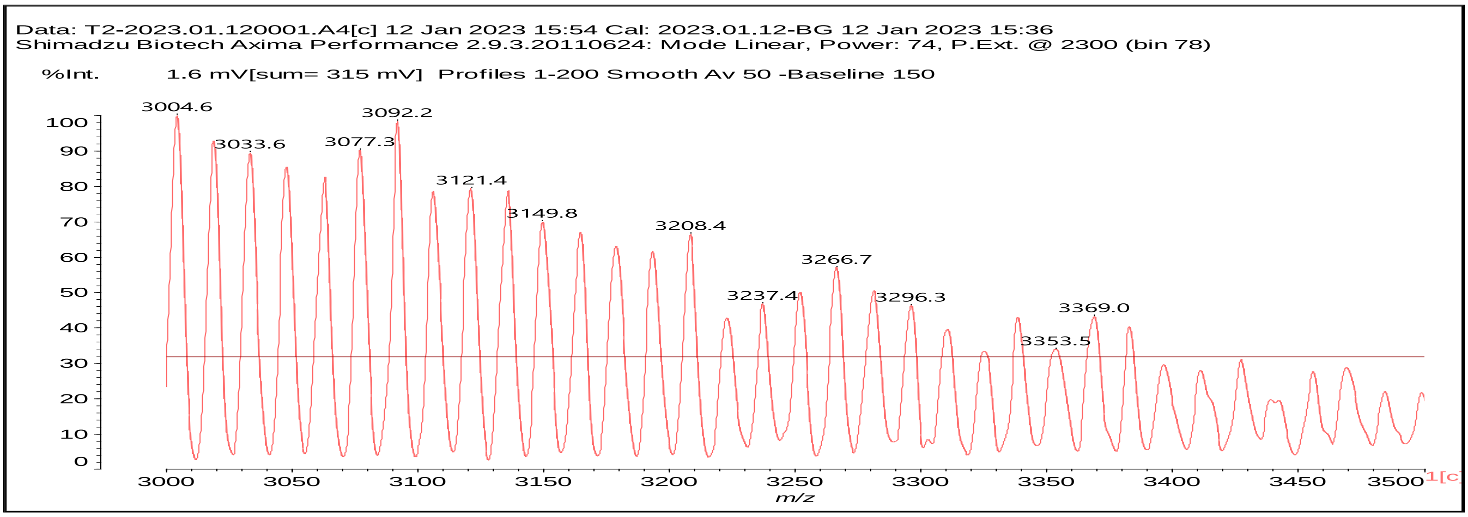

G

Figure S2; A,B,C,D,E,F,G. MALDI ToF Spectra of tannin units linked to hyperbranched tris(2-aminoethyl) amine-urea oligomers

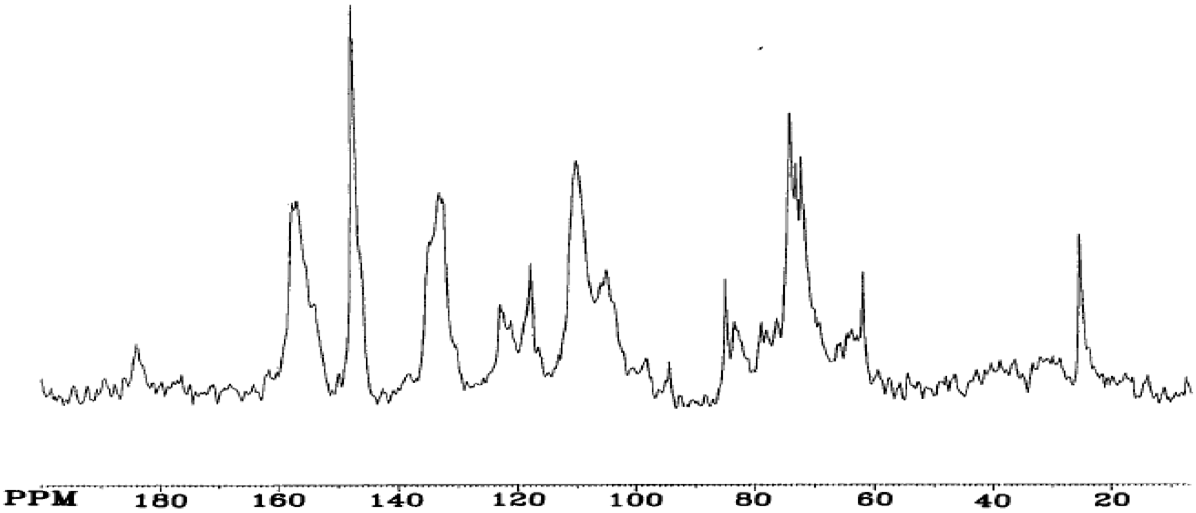

Figure S3. CP MAS ^{13}C NMR spectrum of commercial mimosa condensed tannin extract
